# Mouse Genome Informatics: an integrated knowledgebase system for the laboratory mouse

**DOI:** 10.1093/genetics/iyae031

**Published:** 2024-03-26

**Authors:** Richard M Baldarelli, Cynthia L Smith, Martin Ringwald, Joel E Richardson, Carol J Bult, Mouse Genome Informatics Group, Anna Anagnostopoulos, Dale A Begley, Susan M Bello, Karen Christie, Jacqueline H Finger, Paul Hale, Terry F Hayamizu, David P Hill, Michelle N Knowlton, Debra M Krupke, Monica McAndrews, Meiyee Law, Ingeborg J McCright, Li Ni, Hiroaki Onda, Dmitry Sitnikov, Constance M Smith, Monika Tomczuk, Laurens Wilming, Jingxia Xu, Yunxia Zhu, Olin Blodgett, Jeffrey W Campbell, Lori E Corbani, Peter Frost, Sharon C Giannatto, David B Miers, Howie Motenko, Steven B Neuhauser, David R Shaw, Nancy E Butler, Janice E Ormsby

**Affiliations:** The Jackson Laboratory, Bar Harbor, ME 04609, USA; The Jackson Laboratory, Bar Harbor, ME 04609, USA; The Jackson Laboratory, Bar Harbor, ME 04609, USA; The Jackson Laboratory, Bar Harbor, ME 04609, USA; The Jackson Laboratory, Bar Harbor, ME 04609, USA

**Keywords:** genome informatics, mouse models, genetics, phenotypes, gene expression, model organism

## Abstract

Mouse Genome Informatics (MGI) is a federation of expertly curated information resources designed to support experimental and computational investigations into genetic and genomic aspects of human biology and disease using the laboratory mouse as a model system. The Mouse Genome Database (MGD) and the Gene Expression Database (GXD) are core MGI databases that share data and system architecture. MGI serves as the central community resource of integrated information about mouse genome features, variation, expression, gene function, phenotype, and human disease models acquired from peer-reviewed publications, author submissions, and major bioinformatics resources. To facilitate integration and standardization of data, biocuration scientists annotate using terms from controlled metadata vocabularies and biological ontologies (e.g. Mammalian Phenotype Ontology, Mouse Developmental Anatomy, Disease Ontology, Gene Ontology, etc.), and by applying international community standards for gene, allele, and mouse strain nomenclature. MGI serves basic scientists, translational researchers, and data scientists by providing access to FAIR-compliant data in both human-readable and compute-ready formats. The MGI resource is accessible at https://informatics.jax.org. Here, we present an overview of the core data types represented in MGI and highlight recent enhancements to the resource with a focus on new data and functionality for MGD and GXD.

## Introduction

Mouse Genome Informatics (MGI) is an expertly curated, public knowledgebase of information about the laboratory mouse. It is composed of several interrelated resources, the core being the Mouse Genome Database (MGD) (Blake *et al.* 2021) and the Gene Expression Database (GXD) (Finger *et al.* 2017; Smith *et al.* 2019). MGD curates and integrates information about mouse genes, genomics and gene functions; alleles and their phenotypes; mouse strains and interstrain variation; and mouse models of human disease. GXD curates and integrates expression data from different mouse strains and mutants, with a particular emphasis on endogenous gene expression during mouse development. Both MGD and GXD have been recognized as Global Core Biodata Resources, meeting the criteria of the Global Biodata Coalition for high scientific quality, value, and usage (https://globalbiodata.org/what-we-do/global-core-biodata-resources/). MGI is a founding member of the Alliance of Genome Resources (the Alliance), a confederated union of model organism databases (MODs) (Alliance of Genome Resources Consortium 2022) (https://www.alliancegenome.org/) and the Gene Ontology (GO) Consortium (Ashburner *et al.* 2000; Gene Ontology Consortium *et al.* 2023) (https://geneontology.org). Other resources hosted by and integrated with MGI are the Mouse Models of Human Cancer database (MMHCdb) (Krupke *et al.* 2008), the International Mouse Strain Resource (IMSR) (Eppig *et al.* 2015), and the Recombinase (cre) Data Portal (Perry *et al.* 2022) (https://www.informatics.jax.org/home/recombinase). Together with these affiliated resources, MGI aims to advance our understanding of human health and disease using the mouse as a model system. Here, we provide an update of MGI and highlight some new developments, with a focus on MGD and GXD. First, we report on some MGI-wide improvements that include automating much of our literature acquisition process and a renovation of our Quick Search tool. Then we describe progress in specific biological areas in more detail and highlight some new developments, following a gene to phenotype to disease trajectory. We use corresponding sections of the mouse *Pten* gene detail page as points of reference throughout the narrative. The highlights featured include a novel representation of the pseudoautosomal region (PAR), expanded representation of regulatory features, new cell type annotations for expression data, a new expression profile search tool, and updates to mutation/gene relationships and to the Human–Mouse: Disease Connection (HMDC) resource.

## General procedures, standards, and searching

### Literature acquisition and triage

MGI curates information from relevant peer-reviewed journal articles, which represent a subset of all the articles that report mouse data. The process of collecting MGI-relevant articles has transitioned from a series of manual steps to an automated literature acquisition pipeline. We now search for and download potentially relevant articles from PubMed Central and Science Direct journals, and have implemented a machine learning classification step that fully automates the MGI relevance decision process (see *Methods* for details on our transition to automation). This automated pipeline frees MGI curators of this necessary, but time-consuming task, so more time can be devoted to curating data from MGI-relevant articles. We have observed increased rates of MGI-relevant papers entered into our system, and manually indexed or curated papers, since we implemented the automated literature acquisition pipeline.

### Data and metadata standards

Biological source metadata is notoriously under standardized in public data repositories and in the published literature. For example, in our various data loads that include mouse age designations, we have found 60 differently named database fields reporting the age of samples in one resource alone (Smith *et al.* 2020). In addition, authors often report phenotypic observations in publications using free text descriptions, which makes it difficult to find all relevant publications for a given phenotype from text-based reference searches. MGI specializes in the development and application of data and metadata standards, which are central to our integration process, assure comprehensive database queries, and contribute to MGI's compliance to FAIR principles (Wilkinson *et al.* 2016). MGI uses these standards to annotate and integrate data from the literature, from data loads and from electronic submissions, such that these annotations are reliably searchable. Data standardization methods include incorporating biological ontologies as the framework for annotations and queries; extensive use of controlled vocabularies to systematize source metadata; establishing nomenclature standards throughout MGI; and issuing accession IDs that remain stable through nomenclature transitions. MGD serves the research community as the authoritative data source for nomenclature (of genes, alleles, and strains), and for various annotation types [mouse GO, Mammalian Phenotype (MP) Ontology, and Disease Ontology (DO) annotations] (Table 1). MGD also maintains and distributes the MP ontology (Smith and Eppig 2009). GXD maintains and distributes the Mouse Developmental Anatomy (EMAPA) Ontology and the Adult Mouse Anatomy (MA) Ontology (Hayamizu *et al.* 2015).

**Table 1.** Data types for which MGI is the authoritative source.

Authoritative source services
Unified catalog of mouse genes and genome reatures
Mouse gene and genome feature nomenclature
Mouse allele, transgene and genome rearrangement nomenclature
Mouse strain nomenclature
Mouse Gene Ontology (GO) annotations
Mammalian Phenotype Ontology (MP)
Mouse Phenotype annotations (MP annotations)
Mouse Models of Human Disease (DO annotations)
Mouse Developmental Anatomy Ontology (EMAPA)
Adult Mouse Anatomy Ontology (MA)

Abbreviations	
MGI resources	
MGI	Mouse Genome Informatics
MGD	Mouse Genome Database
GXD	Gene Expression Database
MMHCdb	Mouse Models of Human Cancer database
IMSR	International Mouse Strain Resource

Other resources	
The Alliance	Alliance of Genome Resources
GOC	Gene Ontology Consortium
HGNC	Human Genome Organization (HUGO) Gene Nomenclature Committee
RGNC	Rat Gene Nomenclature Committee
MPD	Mouse Phenome Database
IMPC	International Mouse Phenotyping Consortium
HGVS	Human Genome Variation Society

Tools	
HMDC	Human–Mouse: Disease Connection
MGV	Multiple Genome Viewer
NOCTUA	A collaborative Gene Ontology annotation tool developed by the GO Consortium

Ontologies	
GO	Gene Ontology
MP	Mammalian Phenotype Ontology
HP	Human Phenotype Ontology
DO	Disease Ontology
EMAPA	Mouse Developmental Anatomy Ontology
MA	Adult Mouse Anatomy Ontology
CL	Cell Ontology

Additional abbreviations used frequently	
Build 38	GRCm38 mouse genome assembly (the previous reference assembly for mouse)
Build 39	GRCm39 mouse genome assembly (the current reference assembly for mouse)
PAR	Pseudoautosomal Region
PARX	Pseudoautosomal Region on the X chromosome
PARY	Pseudoautosomal Region on the Y chromosome
TSS	Transcription Start Site

### Searching MGI

MGI provides several different search tools to help users access integrated data from different biological perspectives and tailor search results to the areas most relevant to their interests. General search tools include the Quick Search and several batch query options, while a set of data domain-specific search forms and searchable biological ontology browsers provide for more specialized queries. MGI also hosts two interactive genome browsers and offers programmatic access to most MGI data. Whether users prefer to start with broad, comprehensive queries and then refine result sets progressively, or to compose more narrow, biologically sophisticated queries and then further refine query parameters and/or result sets, MGI's suite of search tools can accommodate these different search strategies.

### General search tools

#### Quick Search

**Fig. 1.**
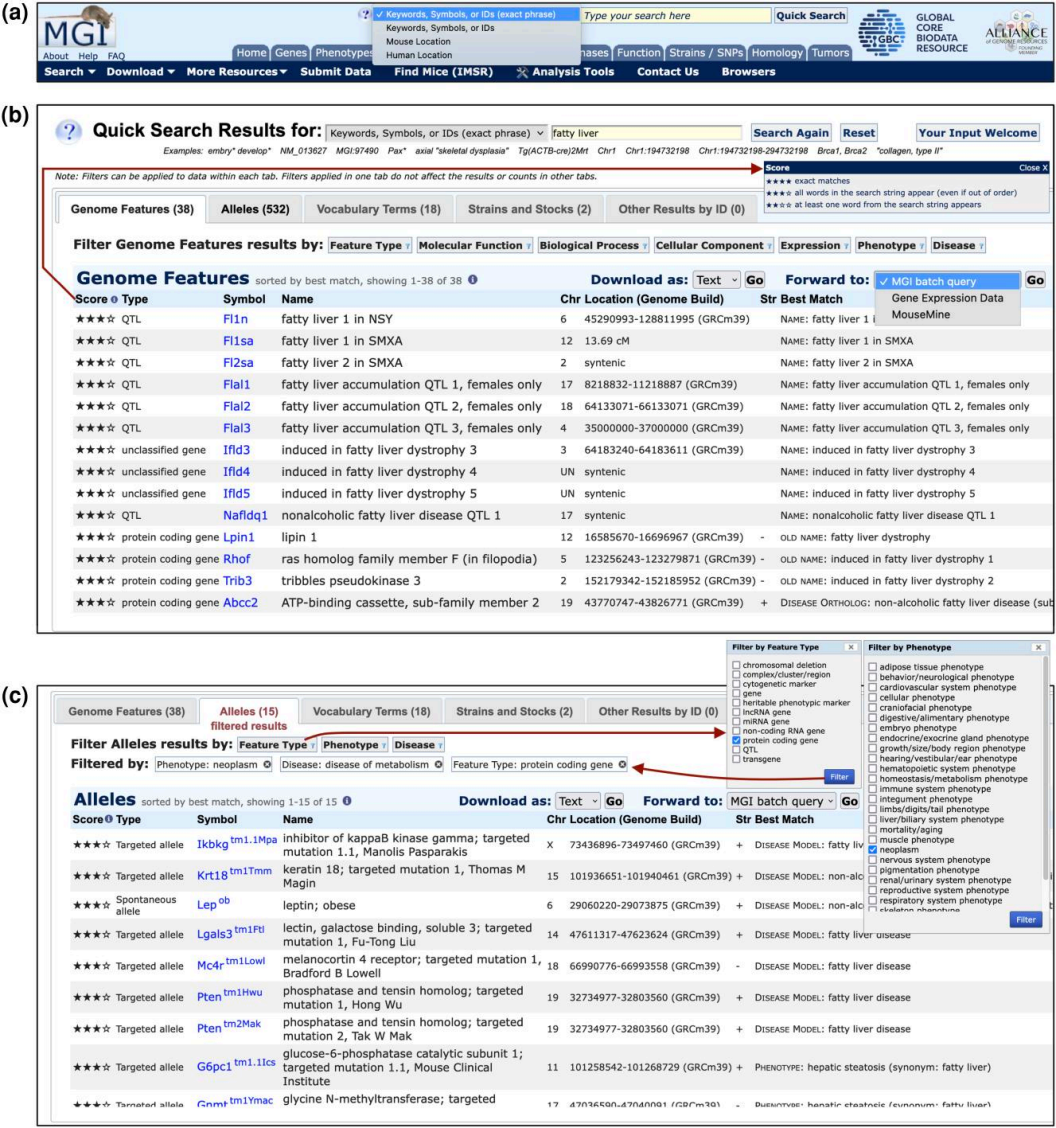
The Quick Search Tool runs five simultaneous searches that provide an onramp to MGI data. a) All MGI pages display the Quick Search input box at top-right of the page. Three search modes are available: search for Keywords/Symbols/IDs that contain the exact phrase of the query (first option, default) or that contain any word of the query (second option), and two location modes that search MGI by genome coordinates (mouse or human). The exact phrase mode is also triggered by enclosing a search string in quotes. The tool reverts to Mouse Location mode automatically if coordinates are entered, unless Human Location mode is selected (not shown). b) Results from an exact phrase search for phrase: *fatty liver* are shown. The first tab containing at least one search result is opened by default (the Genome Features tab in this case). Type, nomenclature, and location for each matching genome feature are provided along with the item associated with the feature that best matches the query (last column) and the score of the match (first column). Star tiers are defined in the Score popup (arrow). Results are sorted first by score, then by the weight of the type of item best matched (current nomenclature > old nomenclature > vocabulary terms) and finally by Symbol, which also links to the corresponding gene detail page. All five tabs are shown, even if no results are returned for a given tab, and result counts from each of the five data area searches are shown in the tabs for those results [Genome Features (38), Alleles (532), Vocabulary Terms (18), Strains and Stocks (2), and Other Results by ID (0)]. Each tab has its own table columns, links to relevant MGI pages, pagination controls and set of biological domain filters. Results from each tab can be exported as plain text or spreadsheet or forwarded in batch to MGI resources for additional information on the whole result set. The Genome Features tab offers the most extensive filter set, including Feature Type, Gene Function, Expression, Phenotype, and Disease options. c) The same search in panel b showing the Alleles tab with filtered results. Filtered tabs are indicated and display the count of filtered results. The original set of 532 alleles from the search for *fatty liver* were restricted to 15 alleles by applying three filters: Feature Types (options shown, execution path shown with arrows), Phenotype (high-level terms of the MP Ontology, options shown), and Disease (high-level terms of the DO, options not shown). Note the filter options for the Alleles and Genome Features tabs are different. The resulting alleles are of protein-coding genes and are associated with neoplasm phenotypes and one or more metabolic diseases (two alleles of the *Pten* gene satisfy these conditions). The Allele tab displays allele type and nomenclature, and allele symbols link to MGI Allele Detail pages. Location information is for associated genes. Score and best match are as described in panel b.

The Quick Search tool is the most commonly used search interface for MGI data. It is prominently featured on the MGI home page (https://www.informatics.jax.org/) and is also available at the top of all MGI webpages (Fig. 1a). The results of a keyword search using the Quick Search provides users an overview of all MGI data types associated with their search terms or accession identifiers (IDs), and provides direct access to MGI details for the search results of interest by performing five separate searches simultaneously against different MGI data types. Separating search results into five separate tabs, the tool returns Genome Features, Alleles, Vocabulary Terms, Mouse Strains and Other Results by ID (homology records, expression assays, sequences, molecular probes, and references), searching MGI nomenclature (gene, allele, strain, etc.), all MGI vocabulary terms (from biological ontologies), and most accession IDs.

We have renovated and modernized the Quick Search tool to improve usability and performance of the interface. The major changes to the tool include (1) using separate tabs to display results from the separate searches instead of showing all result sets on a single page, (2) adding a separate search for mouse strains, (3) adding a default exact phrase search mode, (4) enabling Quick Search queries by mouse or human genome coordinates, and (5) incorporating tab-specific filters to narrow search results by biologically useful criteria relevant to each results tab. Further user interface improvements include scrolling, pagination and result download options on each tab, and forwarding to MGI and GXD batch query resources and to MouseMine on the Genome Features and Alleles tabs. A Quick Search sample query is shown in Fig. 1b, which illustrates these user interface updates.

#### Batch and programmatic access

MGI provides a general batch query tool (https://www.informatics.jax.org/batch), which allows users to search the knowledgebase using a list of gene symbols or identifiers. MouseMine (https://www.mousemine.org/mousemine) (Motenko *et al.* 2015) is another integrated resource of MGI data that is based on the InterMine framework. MouseMine provides diverse query templates, the ability to upload, generate or modify feature lists of different types (genes, alleles, etc.), and also serves as a data warehouse and the primary API for robust programmatic access to MGI.

### Specialized search tools

*Data domain-specific search forms*: Domain-specific search forms are provided that take full advantage of MGI integrated datasets, including mouse orthology data for human, rat, and zebrafish. Integrated search forms are available for genes and other genome features, alleles and phenotypes, recombinase alleles, expression data and images, GXD literature and RNA-Seq and microarray experiment indexes, strains and SNPs, and references. Result summaries from these search forms offer filtering options to help users further refine their result sets. The MGI home page (https://www.informatics.jax.org) is a starting point for access to these forms, and a summary of the forms with links is provided below. See the *Gene expression* section later in this paper for a summary of expression data-specific search forms.

#### Genes and markers query form

Search for genes and genome features by nomenclature, chromosomal location, feature type, and various biological annotation types (gene function, phenotype, disease) (https://www.informatics.jax.org/marker).

#### Phenotypes, alleles, and disease models search

Search for mutant alleles, transgenes, or Quantitative Trait Loci (QTL) variants by nomenclature, chromosomal location, phenotype, disease, or allele categories (https://www.informatics.jax.org/allele/).

#### Human—mouse: disease connection

Search for published and potential mouse models of human disease and candidate genes, and explore phenotypic similarities between mouse models and human patients (https://www.informatics.jax.org/humanDisease.shtml).

#### Recombinase (cre) activity query

Search for recombinase-carrying alleles by recombinase activity profile or by the gene driving recombinase expression (https://www.informatics.jax.org/home/recombinase).

#### MGI strain query

Search for mouse strains by nomenclature, accession ID or strain attributes (https://www.informatics.jax.org/home/strain).

#### Mouse SNP query form

Search for SNPs by associated genes or by genome region (https://www.informatics.jax.org/snp).

#### Reference query form

Search for references in MGI by bibliographic specifications (https://www.informatics.jax.org/reference).

### Searchable ontology browsers

MGI has a set of searchable ontology browsers that are linked to MGI's phenotype, disease and disease model, expression, and functional annotation data (Supplementary Fig. 1). Ontology terms may be searched across the different ontologies via the Quick Search (discussed above) or searched and browsed within each browser itself. The term detail pages include the term name and synonyms, definition, the direct parentage of the term in the ontology, the unique ontology ID and any secondary IDs. The term tree view shows the relationship of the term to all other terms in the ontology. Links following the terms, or data in additional tabs (DO browser), provide access to entities annotated to that term in MGI. Links to MGI's ontology browsers are provided below.

*MP Ontology Browser*: (https://www.informatics.jax.org/vocab/mp_ontology).

*Human Phenotype (HP) Browser*: (https://www.informatics.jax.org/vocab/hp_ontology).

*Disease Ontology (DO) Browser*: (https://www.informatics.jax.org/disease).

*Mouse Developmental Anatomy (EMAPA) Browser*: (https://www.informatics.jax.org/vocab/gxd/anatomy/EMAPA:16039).

*GO Browser*: (https://www.informatics.jax.org/vocab/gene_ontology).

### Interactive genome browsers

#### JBrowse

JBrowse is an open source, interactive genome browser implemented in JavaScript (Skinner *et al.* 2009; Buels *et al.* 2016). MGI supports an instance of JBrowse for interactive viewing of mouse genome annotations in reference genome context, from selectable tracks dedicated to MGI gene model providers and to MGI allele and phenotype data.

#### Multiple Genome Viewer

The Multiple Genome Viewer (MGV) (https://www.informatics.jax.org/mgv/) is a genome annotation visualization tool that allows comparative examination of homologous genome annotations across multiple genomes simultaneously (Richardson *et al.* 2022) (https://www.informatics.jax.org/mgv). Developed to meet the need for a tool to graphically compare genome features across multiple mouse strains, the MGV contains the annotated genomes of 19 mouse strains [the reference mouse genome, 16 inbred strain genomes (Keane *et al.* 2011; Lilue *et al.* 2018), and 2 wild-derived strain genomes (Thybert *et al.* 2018)]. While the MGV remains an important tool for mouse interstrain comparisons, it was designed to display homologous genome features from different species, and currently supports the genomes of all nine species represented in the Alliance of Genome Resources (the Alliance) (Alliance of Genome Resources Consortium 2019).

**Fig. 2.**
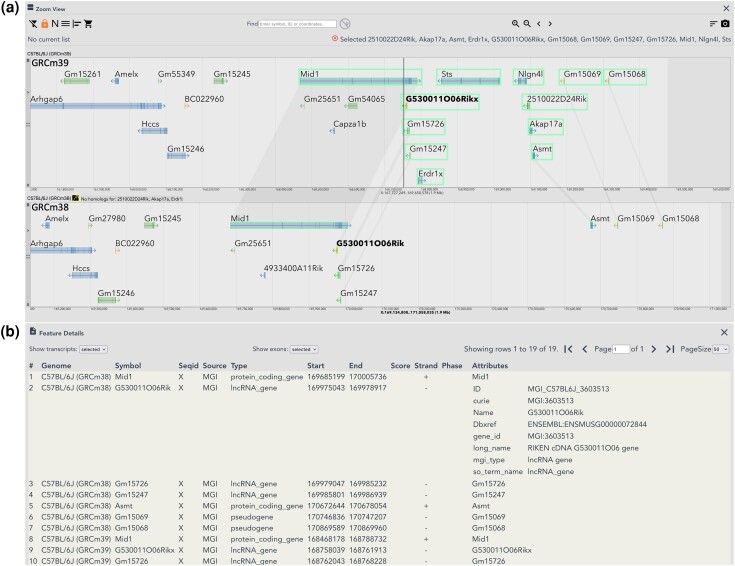
Pseudoautosomal Region (PAR) features in GRCm39. MGV view of the PARX region, comparing GRCm39 vs GRCm38 assembly representations of PARX annotations. a) MGV Zoom View showing distal ends of Chr X in the *C57BL/6J* (GRCm39) (top) and *C57BL/6J* (GRCm38) (bottom) genomes. PARX features in the *C57BL/6J* (GRCm39) genome are boxed with connectors shown to corresponding features annotated in the GRCm38 genome. Annotations for *Erdr1x*, *Sts*, *Nlgn4l*, *2510022D24Rik* and *Akap17a* are only present in GRCm39. A display limit truncates the “No homologs for:” section, but users can scroll through that section in the MGV to see the full list. A vertical line marks the approximate PAR boundary in the Build 39 genome at position chrX:168752755, which falls within the *Mid1* gene in strain *C57BL/6J*. Although transcription direction arrows in the glyphs for some PARX features cross the PAR boundary in the figure (*G530011O06Rikx* and *Gm15726*), Build 39 coordinates for those features are inside the PAR region. b) Feature Details view showing new GFF3 file information for the first 10 rows in the table. The Feature Details table displays all features selected in the Zoom View and their counterparts in other genomes open, sorted by Genome/start coordinate. Mouseover on any row in the table accents in bold the corresponding feature and its counterparts in the Zoom View (mouseover effect on GRCm38 feature *G530011O06Rik* shown). Also shown is expansion of the new Attributes column for row 2 (GRCm38 feature *G530011O06Rik*). Transcripts and exons can be included in the table using the “Show transcript:” and “Show exons:” options above the table.

All MGV datasets have been updated and three new annotated reference genomes have been added: *Xenopus tropicalis* and the large (L) and small (S) subgenomes of *Xenopus laevis*. Also added is the mouse reference genome from the previous assembly (GRCm38) to provide cross referencing between the current (GRCm39) and the previous mouse genome builds, for cases where published data refer to the GRCm38 assembly (Fig. 2). Over 8,000 publications in MGI refer to the GRCm38 assembly searching full reference text. MGV interface improvements include expanding the Feature Details table to show all columns in the corresponding GFF3 files (shown in Fig. 2b).

## Genes and genome features

**Fig. 3.**
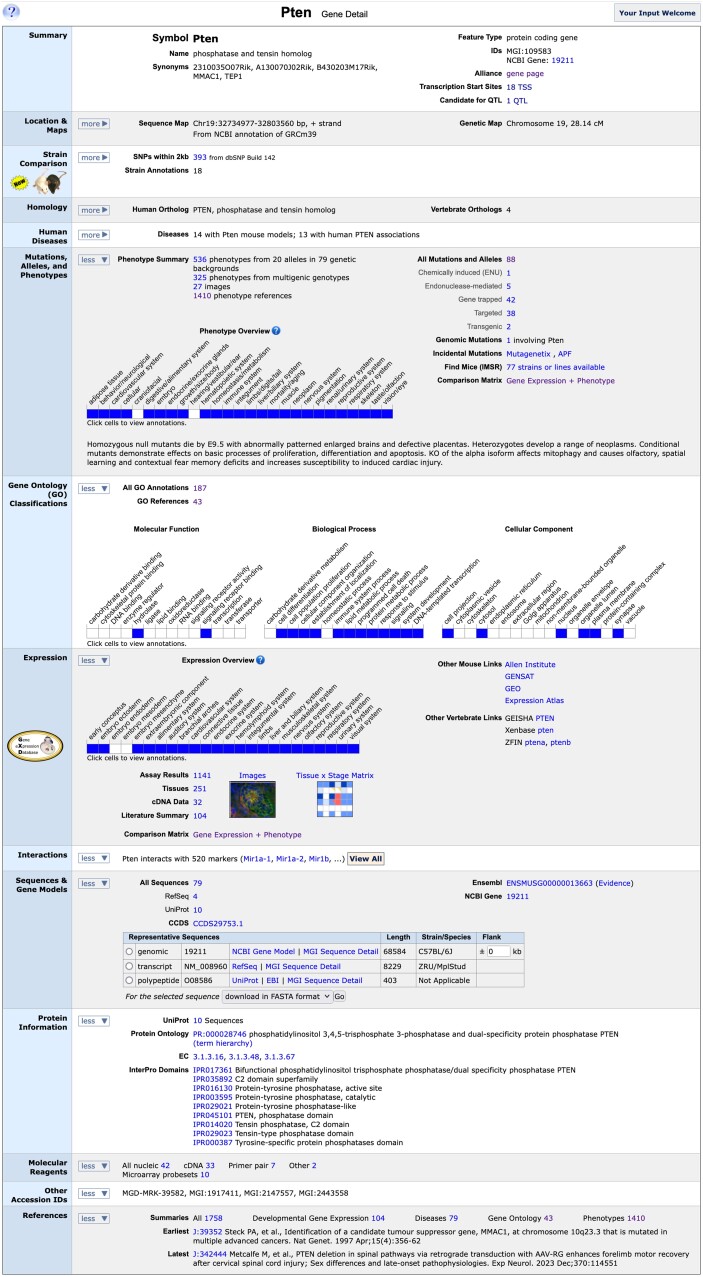
The MGI mouse Pten gene detail page. The mouse gene detail page for Pten shows the integrated at-a-glance summary information associated with the gene in MGI. Summary data shown here is from MGI's curation pipelines, direct data submissions from researchers and integration of data from a large number of external resources. The page is divided into multiple data type sections with links into more detailed information within MGI and to data details at external sites.

Genome features (genes, pseudogenes, QTL, regulatory regions, genetic markers, etc.) are central to the integration of all other data types in MGI and are the primary targets of biological annotations. MGI maintains a comprehensive catalog of mouse genes and other genome features, unified and resolved by connections to genome sequence data and to relevant biological annotations. The current catalog contains over 680,000 genome features covering an array of different feature types that can be positioned in the genome (Table 2). Every genome feature in MGI has a marker detail page, which presents the full range of associated data for the genome feature and serves as an access point that links to further details for each associated data type, including links to corresponding references. The References section at the bottom of the gene detail page provides easy access to all references associated with the corresponding genome feature. The mouse *Pten* gene is a good example of a well annotated gene that, when disrupted, gives rise to mice that model several human cancers and other diseases. Originally identified as the mouse ortholog of human *PTEN*/*MMAC1* (Steck *et al.* 1997), mouse *Pten* encodes a phosphatase with tumor suppressing activity mediated by its role as a phosphatidylinositol 3 kinase (PI3K) signaling antagonist (Stambolic *et al.* 1998; Kim *et al.* 2010; Carnero and Paramio 2014; Xu *et al.* 2014). As we discuss the different data types and connections in MGI, we regularly refer to relevant sections of the *Pten* gene detail page as illustrations of our representation and of how to find corresponding information associated with a given feature (Fig. 3).

**Table 2.** Genome feature types in MGI.

Primary Feature type	Count
Gene	63,750
Noncoding RNA gene	33,907
Protein-coding gene	23,171
Unclassified gene	3,234
Heritable phenotypic marker	2,783
Gene segment	648
DNA segment	20,382
Pseudogene	15,940
Transgene	11,684
QTL	8,496
Cytogenetic marker	1,373
BAC/YAC end	887
Complex/Cluster/Region	236
Other Genome Feature	559,364
TSS cluster	164,748
Enhancer	154,792
CTCF binding site	110,891
Open chromatin region	61,804
Promoter	25,213
CpG island	23,022
Transcription factor binding site	17,329
Additional features in this category	1,565

Counts are shown for primary feature types (left justified) and for subcategories of Genes and of Other Genome Features (indented).

### Gene nomenclature, synonyms, and identifiers

As the central repository for official mouse gene symbols and names, we use standardized nomenclature throughout MGI, following guidelines of the International Committee on Standardized Genetic Nomenclature for Mice. We coordinate ortholog gene naming conventions with the Human Genome Organization (HUGO) Gene Nomenclature Committee (HGNC), and with the Rat Genome Nomenclature Committee (RGNC). The unique symbol and name of any gene or genome feature can be found at the top of the Summary section of the gene detail page for that feature, along with former symbols, synonymous terms and a feature type classification (see *Methods* for MGI feature type details). To assure productive queries by outdated nomenclature or by other names used in the literature for the same feature, we maintain a searchable inventory of withdrawn symbols and synonymous terms for each genome feature. Synonyms in MGI are curated from the literature, imported from NCBI gene, and submitted from researchers. MGI genome features are uniquely and stably accessioned and the primary MGI identifier is listed on gene detail pages along with the NCBI gene ID if available. Secondary IDs for a genome feature, which are generated from gene merges and other nomenclature events are searchable and are displayed in the Other Accession IDs section of the gene detail page, near the bottom of the page.

### Alliance of genome resources

MGI provides a link to the corresponding gene detail page at the Alliance of Genome Resources when available. Additional content at the Alliance includes Paralogy, Pathways, Interactions and Variant data, some of which is curated by MGI for display at the Alliance.

### Relationships between genome features

MGI uses special relationships between genome features to represent important reference-supported biological concepts, such as transcription initiation of associated genes from defined transcription start site (TSS) features, candidate genes for QTL, reported interactions between QTL, and the connection between homologous PARX and PARY features in the PAR. When a genome feature has any of these relationship types, they are displayed in the bottom-right corner of the Summary section on corresponding gene detail pages. All genome feature relationship links are reciprocal and provide relationship details. See *Methods* for more details on Transcription Start Sites, Candidate for QTL, and Interacting QTL relationships. The Homologous PAR Feature relationship is discussed in detail in the *Pseudoautosomal region* section below.

### Genomic and genetic map locations

**Fig. 4.**
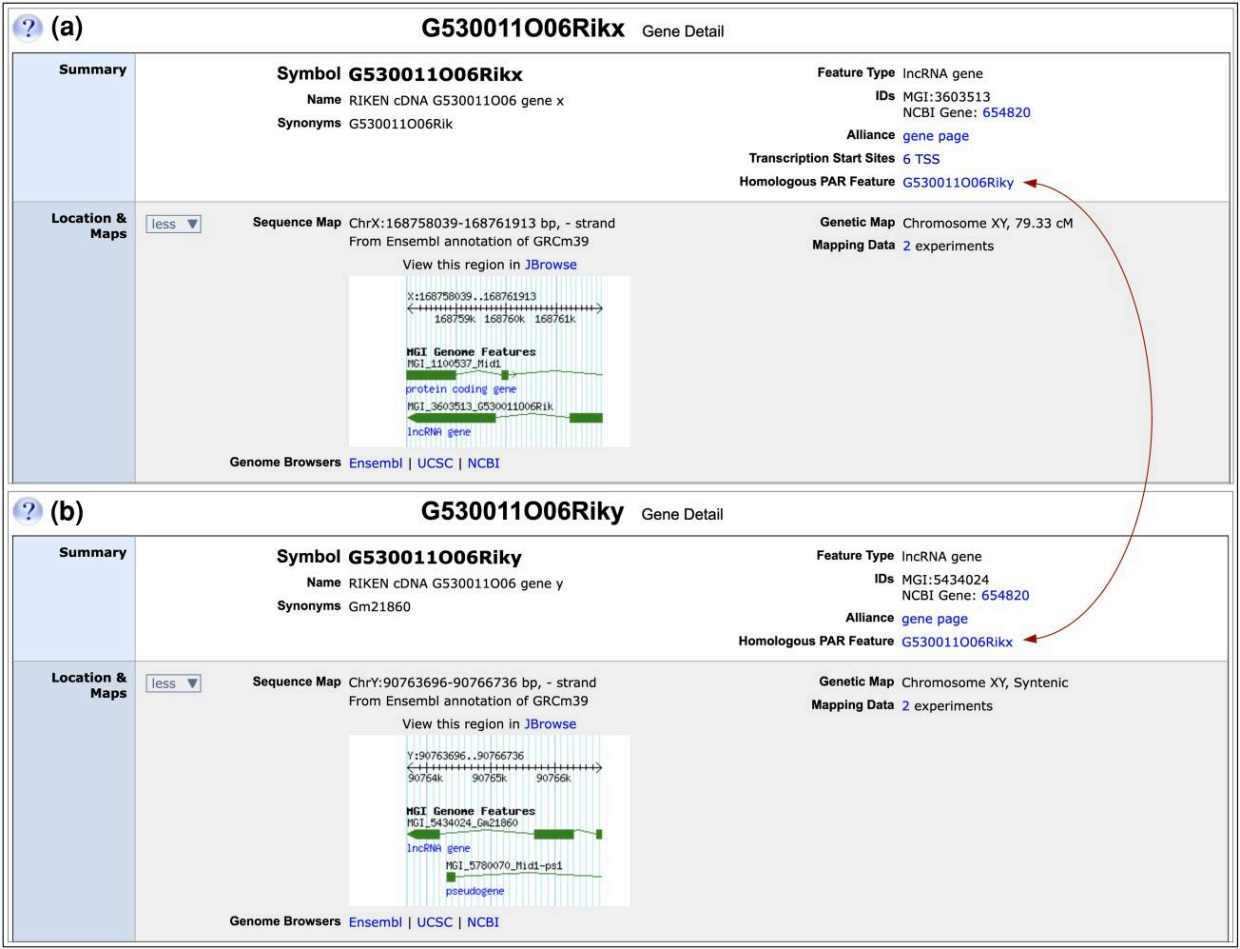
Representation of PARX/PARY partners in MGI. The first two sections of the gene detail pages for PARX/PARY partners, *G530011O06Rikx* and *G530011O06Riky* are shown. a) Partial gene detail page for *G530011O06Rikx*. In the Summary section, gene nomenclature has suffix “x”, indicating the feature is a PARX partner (X chromosome homolog). Synonyms show gene nomenclature from Build 38 annotation. In the right column of the Summary section, the NCBI Gene ID links to the corresponding NCBI Gene record that represents both PARX and PARY homologs. In the new “Homologous PAR Feature” subsection, a reciprocal link is provided to the gene detail page for the PARY partner (*G530011O06Riky*) shown in panel b (arrows). The Location & Maps section shows genome coordinates on the X chromosome in the Sequence Map subsection and chr XY as the chromosome in the Genetic Map subsection. Links are provided to corresponding X chromosome regions in various genome browsers and a thumbnail view of the region is shown that links to JBrowse. The Genetic Map subsection has links to legacy mapping experiments for this gene, which are the source of its cM position. b) Similar view of the gene detail page for *G530011O06Riky*. The “y” suffix in gene nomenclature indicates the feature is a PARY partner (Y chromosome homolog), and previous (Build 38) gene nomenclature is shown under Synonyms. *G530011O06Riky* links to the same NCBI Gene record as does *G530011O06Rikx* (654820), and a reciprocal link to the PARX partner (*G530011O06Rikx)* is provided in the Homologous PAR Feature subsection (arrows). The Sequence Map subsection of *G530011O06Riky* shows genome coordinates from the Y chromosome, and chr XY as the Genetic Map chromosome.

MGI stores genetic map locations and genome coordinates for genome features, both of which are displayed in the Location & Maps section of gene detail pages. Coordinates for a genome feature are displayed in the Sequence Map subsection with strand, genome build and the coordinate provider. Features with no coordinates display a note to that effect in this section. When the Location & Maps section is toggled open, links are provided to the corresponding feature in genome browsers (JBrowse, Ensembl, UCSC, and NCBI's new Genome Data Viewer). The Genetic Map subsection shows the genetic map chromosome and cM and/or cytoband positions (if available), and when the section is toggled open, displays the number of associated genetic mapping experiments with a link to mapping experiment details. For the vast majority of features in MGI, the Sequence Map and Genetic Map chromosomes are the same. The exceptions are genome features in the PAR, which can have genome coordinates on either the X or Y chromosome (Sequence Map) and have chr XY as the Genetic Map chromosome (Fig. 4).

### Sequences & Gene Models

The Sequences & Gene Models section of the gene detail page provides access to all genomic, transcript, and protein sequences associated with the gene or genome feature via links to several Sequence Summary pages: one for all associated sequences and separate summary pages for associated NCBI RefSeq and UniProt sequences. Sequence Summary pages list details and provide links to more information for each associated sequence (including FASTA sequences), and have the option to filter by sequence type, which is useful when many sequences are involved (*Pten* sequence summary page link: https://www.informatics.jax.org/sequence/marker/MGI:109583). The Sequences & Gene Models section also provides links to the Consensus CDS (CCDS) project (Pruitt *et al.* 2009), the Ensembl gene summary view, and another link to the corresponding NCBI Gene record for the featured gene. Well-studied genes such as *Pten* have many associated sequences.

### Representative sequences

MGI uses the concept of a representative sequence for each sequence type (genomic, transcript and protein) as a means of providing users easy access to a single, symbolic sequence of each type. In most cases (see *Methods* for exceptions), the genome coordinates of the representative genomic sequence also serve as the coordinates for the genome feature itself, and are used in coordinate-based searches and in coordinate displays for the feature throughout the MGI interface (see Location & Maps section in Fig. 3). Representative sequences are selected using rule-based algorithms that take into account sequence providers, lengths, and overall sequence quality for each sequence type associated with the genome feature (see *Methods*). The Representative Sequences table in the Sequences & Gene Models section of the gene detail page displays the MGI-designated representative genomic, transcript and protein (if protein-coding) sequences of the associated feature. Links to provider details and to MGI sequence detail pages for each representative sequence type are provided along with sequence length and mouse strain information. FASTA sequence files for any of the representative sequences can be downloaded (or sent to NCBI BLAST), and for the FASTA file download of representative genomic sequences, an option is provided to add a user-defined nucleotide flank (in kb) to the downloaded sequence (see Fig. 3).

### Build 39 update

The mouse research community has benefited from a quality reference genome assembly for over a decade. The GRCm38 assembly [GCF_000001635.20 (Build 38)] was stable and useful, but contained a number of problematic regions that the Genome Reference Consortium (GRC) has worked to resolve in its 2020 release of the GRCm39 assembly [GCF_000001635.27 (Build 39)], which is the latest version of the mouse reference genome assembly (strain C57BL/6J), and is the first coordinate-changing update since Build 38 was released in 2012. Build 39 contiguity increased significantly compared to Build 38 (scaffold N50 increased by 95%) and the gap count dropped by 45%. In addition, over 370 reported issues were resolved, which resulted in a drop in the number of regions with patches/alternative loci by 96%. Genome coordinate annotations have been updated to the GRCm39 assembly throughout MGI. This includes all genome features, alleles and variants with coordinates, and various notes fields that contain genome coordinates embedded in note text. See *Methods* for details on the update to Build 39.

### Homology in MGI

Vertebrate orthology is used extensively in MGI for cross-species searches, conserved etiological assertions for the HMDC and the DO Browser, and to indicate and standardize orthologous components used in allele constructs. MGI loads stringent mouse ortholog data for human, rat, and zebrafish from the Alliance, and in the Homology section on gene detail pages summarizes ortholog data for the gene (Fig. 3). When this section is toggled open, additional information is provided for human orthologs and for the stringent ortholog cluster from the Alliance. A link is provided to the MGI Vertebrate Homology detail page, which provides additional details for all orthologs in the MGI cluster (Supplementary Fig. 2) (*Pten* Vertebrate Homology page link: https://www.informatics.jax.org/homology/cluster/key/45855675). Links are also provided to corresponding superfamily data at the Protein Information Resource (PIR), and to Ensembl's Gene Tree, a phylogeny-based homology resource.

### Molecular reagents for genome features

Molecular clones and probes were the bedrock of the recombinant DNA revolution and accelerated the field into the modern age of whole genome, next generation technologies. PCR-generated probes remain prolific in the literature for spatial resolution of gene expression using in situ techniques. MGI represents modern and legacy nucleic acid reagents used in published gene expression and genetic mapping studies of mouse genes and genome features. For many mouse genes, the original molecular clones are among the MGI molecular reagents for the genes. A summary of molecular reagents in MGI for each gene can be found in the Molecular Reagents section on gene detail pages. Links are provided to summary pages with additional information for all reagents associated with the gene and for subsets distinguished by nucleic acid type [genomic, cDNA, primer pairs, and other reagents (such as oligonucleotides)]. The *Pten* gene has 42 associated molecular reagents, most of which are cDNA clones. Also included in the Molecular Reagents section are links to a summary of the Affymetrix microarray probe sets that include probes for the featured gene on three common mouse Affymetrix microarray platforms.

### New developments for MGI genome features

#### The PAR in Build 39

Improvements to the Build 39 assembly include a retiling of the PAR on the X chromosome (PARX). The PAR is a short region of homology located at the distal tips of the X and Y chromosomes that mediates proper pairing and segregation of sex chromosomes in males and is the site of meiotic recombination between X and Y chromosomes (Perry *et al.* 2001; Raudsepp and Chowdhary 2016; Dumont 2017). Although the PARX assembly in Build 39 is not complete, several PAR genes missing from the Build 38 assembly are present in Build 39 (Fig. 2). Unfortunately, the Y chromosome assembly in Build 39 remains unchanged from Build 38.

##### Representation of PAR genome features in MGI

With an improved (albeit incomplete) assembly of the PARX region in Build 39, we sought to increase user access to PAR genome features, particularly PARX/PARY homologs. Corresponding PAR features on X and Y chromosomes are homologous alleles (as at any autosomal locus), however, representing PARX/PARY homologs as alleles in MGI presents a challenge to our integrated search paradigm. Searching by genome location throughout the MGI interface is based on the locations of genes and genome features (chromosome + genome coordinates or map position). MGI does not provide coordinate-based searches at the allele level. In addition, the MGI schema allows for only a single set of genome coordinates per genome feature, which is a problem for the one gene/two coordinates model required to accommodate X and Y chromosome alleles for a PAR gene. Furthermore, gene-level annotations such as GO terms are not directly associated with alleles in MGI, which complicates the search for and display of PARX- or PARY-specific data in a PAR allele model. Therefore, to open PARX and PARY components to the full range of MGI's integrated queries, we opted to represent PARX and PARY components as separate genome features, distinctly named and accessioned, and with either X chromosome or Y chromosome coordinates, respectively. MGI's representation of PAR features differs from that of NCBI's, which uses the single gene/multiple coordinates model. NCBI Gene ID: 654820 (NCBI gene symbol: *G530011O06Rik*), for example, lists coordinates on both the X and Y chromosomes.

##### Searching for PAR features

Since the PAR is a valid linkage group, XY is a chromosome on the genetic map and is an option for location-based searches throughout MGI. To allow users the option to search for PAR features comprehensively (by selecting chrXY in location-based searches), we assigned genetic map chromosome XY to all genome features in the PAR. Some PAR features that were mapped to chrXY previously by legacy linkage or cytogenetic mapping studies had already been assigned genetic map chromosome XY. All genome features with Build 39 coordinates that fall within the coordinate ranges for the PARX and PARY regions defined in assembly GCF_000001635.27 were also assigned genetic map chromosome XY. On PAR gene detail pages, both map locations are displayed in the Location & Maps section (Fig. 4). Searches by chrXY (without coordinates) will include all PARX and PARY features with X or Y chromosome coordinates and the set of legacy loci mapped to the PAR that were never assigned genome coordinates. Users can also search for PARX or PARY features within a genome coordinate range, by selecting either the X or Y chromosome and entering the coordinate range of interest for the specified chromosome. In the Build 39 assembly, the PARX region is defined by coordinate range: chrX:168752755-169376592, while the PARY region is defined by coordinate range: chrY:4072168-4161965 (GCF_000001635.27).

##### PARX/PARY homologous pairs

The improved Build 39 assembly and associated gene model annotations allow for limited identification of corresponding PARX/PARY homologs (partners), some of which preexisted in MGI as unrelated genes on chrX and chrY, respectively. To help users identify corresponding PARX and PARY partners, we adopted a new nomenclature convention, where a root gene symbol is shared between the partners followed by an “x” or “y” suffix, for the corresponding chromosome. We also provide reciprocal links between partner PARX and PARY genes in a new “Homologous PAR Feature” section of PAR gene detail pages (Fig. 4). For example, the *G530011O06Rikx* gene (MGI:3603513), which had symbol “G530011O06Rik” in Build 38, is the PARX partner of *G530011O06Riky* (MGI:5434024), which had symbol “Gm21860” in Build 38 (Fig. 4). This special representation of PARX/PARY partners is only done in cases where both the PARX and PARY genes have gene models from either NCBI or Ensembl, and partner gene model sequences are nearly identical. Due largely to a low-quality assembly of the mouse Y chromosome (particularly in the PARY region), we can define only two pairs of PARX/PARY partners at this time (*Erdr1x*/Erdr1y and *G530011O06Rikx*/*G530011O06Riky*), however, we expect to extend this representation throughout the mouse PAR with improvements to the Y chromosome assembly from long-read platforms.

While improvements in the Build 39 assembly of the X chromosome PAR (PARX) are significant, a high density of repetitive sequences in the PAR (Takahashi *et al.* 1994) presents a challenge to short-read-based assemblies. This is reflected in the large sequence gaps interspersed throughout the Build 39 PAR regions. A preliminary effort to sequence through the complete C57BL/6J PAR using a long-read-based primer walking strategy produced a draft sequence of the entire C57BL/6J PAR that is ∼700 kb in length (Kasahara *et al.* 2022). These authors characterized 3 additional PAR protein-coding genes and 3 pseudogenes that are not annotated in Build 39 nor present in MGI, and described a series of polymorphic, tandem segmental duplications at the PAR boundary. MGI will incorporate these additional PAR genome features into our gene unification process once they occupy coordinate space within the reference mouse genome assembly. Improved representation of the PARY region in the reference genome assembly awaits a robust, long-read assembly of the mouse Y chromosome as was done recently for the human Y chromosome (Hallast *et al.* 2023; Rhie *et al.* 2023).

#### Regulatory features in MGI

We recently extended the MGI genome feature catalog to include putative regulatory features annotated to the mouse genome from the Ensembl Regulatory Build (Zerbino *et al.* 2015), the VISTA Enhancer Browser (Visel *et al.* 2007), and from NCBI's RefSeq Functional Elements (RefSeqFEs) (Farrell *et al.* 2022). The mouse Ensembl Regulatory Build standardizes whole-genome epigenomic datasets from the ENCODE Project (Encode Project Consortium *et al.* 2020, 2022) and implements a uniform annotation pipeline that predicts regulatory element types (promoters, enhances, CTCF binding sites, unspecified transcription factor binding sites, and open chromatin regions) and computes activity states in various cell types for the genome regions defined (Zerbino *et al.* 2015). We loaded over 360,000 Ensembl regulatory features with coordinates (from Ensembl 108), which represent the vast majority of regulatory features in MGI (Table 3). We also loaded over 1,300 regulatory features from the VISTA Enhancer Browser (with updated Build 39 coordinates) and nearly 4,000 RefSeqFEs elements from NCBI annotation release: GCF_000001635.27-RS_2023_04 (Table 3). Although smaller datasets than the Ensembl Regulatory Build, the VISTA and RefSeqFEs elements have experimental justification. VISTA enhancers are supported by reporter expression data in transgenic mice (Visel *et al.* 2007), and RefSeqFEs elements are literature-characterized nongenic regions with experimental validation (Farrell *et al.* 2022). While we do not load activity states (Ensembl) or expression data (VISTA/RefSeqFEs) for regulatory features, we link to details for each feature at the respective resource sites from the Other Database Links section of regulatory feature detail pages. Ensembl Regulatory features and RefSeqFEs elements are updated in MGI in conjunction with new Ensembl Regulatory Build and NCBI gene model releases, respectively. VISTA enhancer elements will be updated periodically, when new mouse data are available. In general, regulatory region features in MGI follow the nomenclature convention: symbol = Rr#, name = regulatory region # (Rr1002, regulatory region 1002, for example). Users can search for regulatory features in MGI by feature type, genome coordinate range or by identifiers from the corresponding resources.

**Table 3.** Regulatory features in MGI.

Regulatory Feature type	Total regulatory features	Ensembl regulatory features	VISTA enhancer elements	NCBI RefSeqFEs Elements	Features from literature Curation	Features with mutant alleles	alleles
Enhancer	154,792	149,202	1,338	3,911	341	183	294
CTCF binding site	110,891	110,887		0	4	17	19
Open chromatin region	61,804	61,800		4	0	0	0
Promoter	25,213	25,131		57	25	7	12
Transcription factor binding site	17,329	17,318		0	11	10	12
Additional regulatory feature types	92			15	77	50	121
Totals	370,121	364,338	1,338	3,987	458	267	458

Total distinct regulatory feature counts are shown for each feature type next to corresponding feature counts from regulatory feature loads (Ensembl, VISTA, NCBI) and from literature curation. About 600 enhancer features from the NCBI RefSeqFEs set overlap with VISTA enhancers and are not included in the NCBI RefSeqFEs enhancer count. Counts of regulatory features with mutant alleles in MGI are shown for each regulatory feature type next to the corresponding number of alleles. Compared to other regulatory feature types, a greater number of enhancer elements have experimental support, which is reflected in the counts of MGI-curated data types for enhancers (last 3 columns). That the number of regulatory features created from literature curation matches the number of curated alleles of regulatory features at the time these counts were taken is coincidental.

## Alleles, phenotypes, and strains

MGI maintains the comprehensive catalog of mouse alleles, mutations and strains. Data are incorporated from expert literature curation, small- and large-scale mutagenesis projects, strain and mouse stock centers and repositories, and individual contributions. Despite significant large-scale mutation-generating projects such as the International Mouse Phenotyping Consortium (IMPC) (Groza *et al.* 2023), which created knockout alleles in a large number of genes for study of gene function, individual investigators and rare disease centers continue to create many of their own new specific mouse mutations, such as point mutations mimicking human disease variants, for further in-depth studies of gene function related to disease, or for studies involving genetic interactions, strain-specific modifier or epigenetic effects. MGI collects and integrates these data from multiple sources into a unified catalog. The phenotypic consequences of all of these mutations in mice are described using the MP Ontology (Smith and Eppig 2009) and associated with human disease terms from the DO (Schriml *et al.* 2019) to enable consistent searching and data retrieval across all mutation types. MGI currently holds over 100,547 alleles present in mice which have been used to investigate phenotypes and human diseases in over 72,989 genotypes (Table 4).

**Table 4.** Allele, phenotype, and disease data in MGI.

Mutant alleles cataloged in MGI	691,056
Mutant alleles in mice	100,547
Genes with mutant alleles in mice	17,525
Transgenes and other complex mutations	11,789
Genotypes with phenotype annotations	72,989
Genotypes with disease annotations	7,749
Markers with phenotype annotations	16,110
Markers with disease annotations	3,210

Numbers of mutant alleles in MGI and the numbers of alleles and MGI markers associated with disease and phenotype information.

MGI annotates phenotypes to defined genotypes carried on various mouse strain or hybrid backgrounds using the MP. Each genotype can display an array of different phenotypes. For example, different mutations in the *Pten* gene illustrate the complex relationship to mutation, phenotype and disease (Fig. 3). Clicking on the link for “All Mutations and Alleles” shows an allele summary page for all alleles and mutations affecting the *Pten* gene (not shown). Of the 88 different mutations affecting this gene, *Pten* has 43 different mutations and alleles that have been made into mice, 20 of which have reported phenotypes which are distinct from each other depending on the nature of the mutation, and phenocopy different human diseases related to the human *PTEN* gene as shown in the “Abnormal Phenotypes Reported in these Systems” and “Human Disease Models” columns. High-level summary phenotype data shown on this mutation summary page are gathered from the phenotype-genotype data when the genotype is not complex, i.e. involves a mutation in a single gene. An additional 45 mutations in *Pten* are gene trapped from various resources, or from the International Knockout Mouse Consortium and only exist as ES cell lines as indicated in the Category column.

### Gene-specific mutation, alleles, and phenotypes summary data

The Mutations, Alleles, and Phenotypes section of the gene detail page (Fig. 3) shows a summary view of the mutations and alleles involving the gene. These include mutations in the gene, transgenic mice expressing the gene and genomic mutations such as deletions, inversions and translocations that involve the gene. The number of mutations cataloged are listed and linked to a summary page showing each mutation and summary information about that mutation. Expertly curated high-level qualitative phenotype data associated with these mutations are summarized at a glance in a ribbon display format with more details available by clicking boxes in the ribbon. At the bottom of the section, a human-readable text summary of phenotypes associated with mutant genotypes of the gene is presented.

### Alleles and phenotypes

**Fig. 5.**
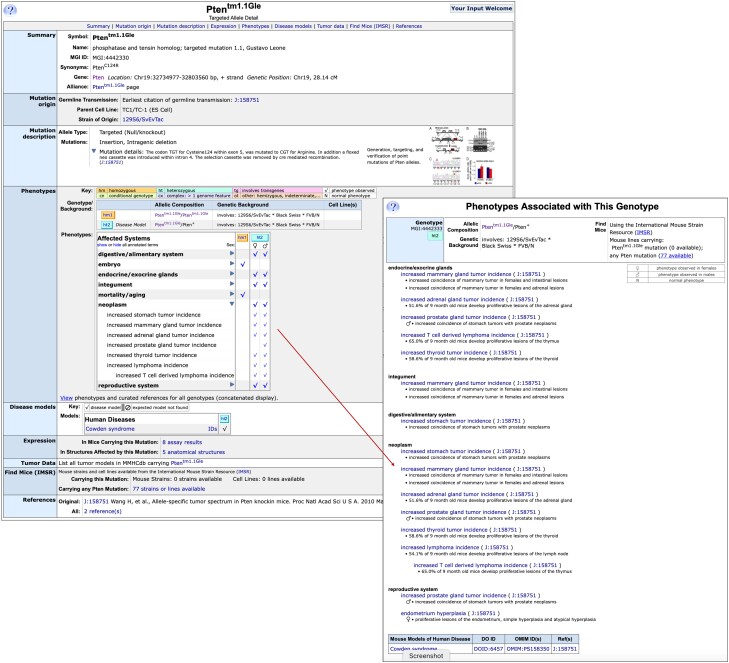
MGI allele detail age for *Pten^tm1.1Gle^*. Information available about the allele includes nomenclature, mutation origin, project collection, and molecular details, when available, phenotype data, IMSR data for location of this mutation in a public repository, references. The phenotype details can be viewed by clicking on the toggles next to the high-level phenotype terms. Shown is the open toggle for “neoplasm.” Checkmarks for annotations to that term are provided to show easy comparison among the different genotypes involving the allele. Clicking the checkmarks will open a popup that shows the term, supporting references and annotation details including sex and genetic background effects if any.

MGI has cataloged over 691,056 spontaneous, induced and engineered mutant alleles, QTL, transgenes and complex mutations in mice and ES cells to date. Information provided on an Allele Detail page (Fig. 5) for each of these mutations includes a summary section with the mutation symbol and name, the MGI unique identifier, synonyms, associated gene(s), and links to the Alliance of Genome Resources allele detail page. Phenotype images associated with the allele from publications may also be shown in the summary section and are included with permission of the author or publisher. The mutation origin and description sections contain the strain of origin, any cell line information, the allele type and summary mutation and a detailed text description. Molecular images illustrating the mutation detail may be shown here, if available. Not currently shown are curated variants for selected alleles. These curated data are available from the Alliance allele detail page linked in the summary section. The mutation description section will also contain allele–marker relationships for certain allele types (see *Allele–marker relationships*).

Phenotypes curated to genotypes involving the mutation are listed in summary format in the Phenotypes section of the allele detail page. The genotype matrix presents a mutant allele in its curated contexts, including a single gene mutation studied in one or more genetic strain backgrounds and more complex genotypes including conditional genotypes, multigenic mutations, transgenic models, or combinations of these. The Genotypes table shows the allele composition and strain genetic background for each genotype and indicates whether a disease model annotation is included. The Genotype boxes or a checkmark within the matrix may be clicked to open a more detailed view that includes phenotype terms, curator notes, and links to references. This link opens in a separate page to facilitate comparison of multiple genotypes. Within the matrix, a high-level summary term in the left column may be toggled open to reveal more detailed phenotype terms for comparisons among genotypes within the matrix view. The link under the matrix opens a new window showing the full text annotations for all genotypes in the matrix.

Diseases associated with genotypes involving the allele are shown in the Disease models section and are associated with the genotypes as in the Phenotypes section. The disease name links to the disease ontology term detail page and other IDs for these diseases are shown by clicking the IDs link. Additional sections include links to GXD expression data via anatomy term in the Mouse Developmental Anatomy Browser when available, to tumor data involving the allele at the MMHCdb (Krupke *et al.* 2008), and links to mouse strain availability for strains carrying this mutation or other mutations in the same gene at the IMSR (Eppig *et al.* 2015). Finally, a list of selected references involving this allele is available at the bottom of the allele detail page.

### Regulatory feature alleles

A new focused curation effort is underway to capture the molecular and phenotypic details of literature published mutations of regulatory regions. Where it is possible to correlate published mouse regulatory mutations with existing regulatory region markers in MGI we do so (Supplementary Fig. 3), otherwise we create new regulatory region markers from such studies. To date, more than 450 mutant alleles of 267 defined regulatory regions have been cataloged and annotated with phenotypes and disease model data when appropriate (Table 3).

### Allele—marker relationships

We recently formalized our relationship between mutations and the markers affected by these mutations, ectopically expressed genetic components such as genes and the features that regulate the expression of the mutation, particularly in transgenes and expression constructs inserted into endogenous loci.

#### Mutation involves

**Fig. 6.**
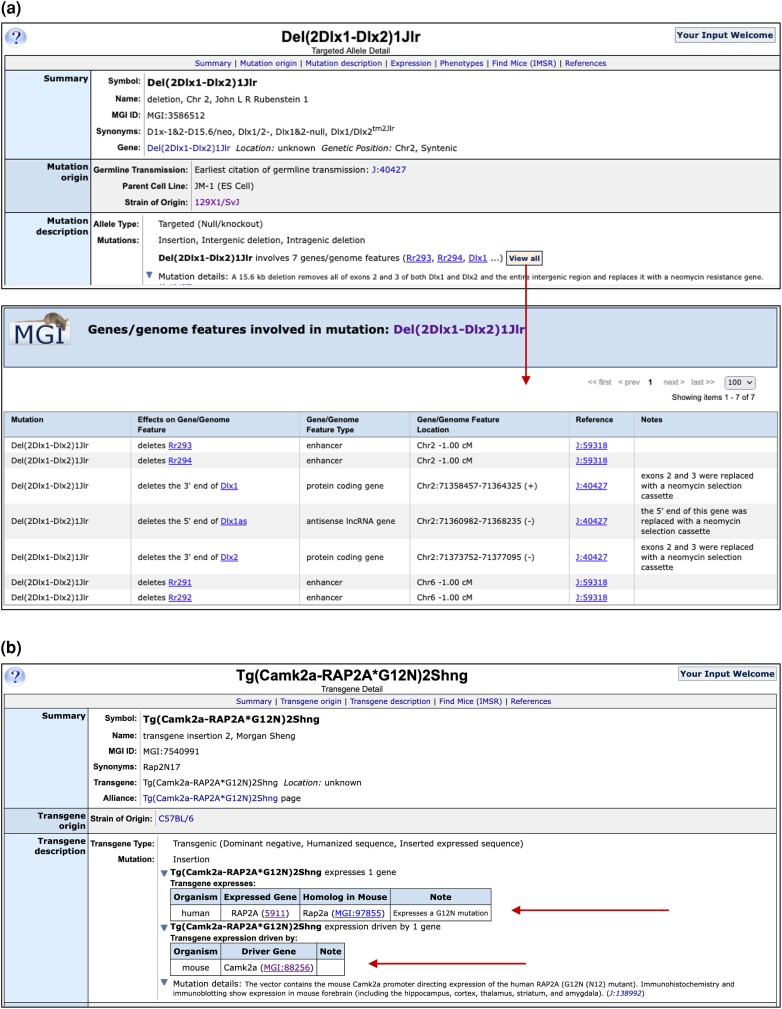
MGI allele relationships to genetic markers. a) Marker relationships are shown in the Mutation Description section of allele detail pages and on allele summary pages. Shown are the Markers related to the deletion mutation Del(2Dlx1-Dlx2)1Jlr curated in MGI. Clicking the “View All” link will open a popup box showing details of the relationship of an MGI marker to the genetic mutation along with supporting references and curator notes. b) Exogenous genes and regulatory elements contained in inserted constructs in Tg(Camk2a-RAP2A*G12N)2Shng mice are shown in the Mutation Description section of allele detail pages. The human RAP2A mutant expressed gene and the mouse Camk2a driver are listed (arrows) and linked to the human gene record at NCBI and to MGI, respectively.

When a mutation affects more than one gene or marker, a link in the allele summary page and on the allele detail page will show all the known genetic entities affected, with links to those entities and the type of relationship (Fig. 6a). These allele–marker relationships are annotated using a controlled vocabulary that includes types of deletions, inversions, duplications, and gene fusions. Functional consequences such as “decreased translational product level” are also used in cases of hypomorphic mutations. These data are available in the MouseMine interface for download and analysis.

#### Inserted expressed sequence including expression of non-orthologous genes

We have shown exogenous expressed sequences on allele detail pages when an ortholog exists for that gene in mouse. We now show NCBI or MGI links for all expressed genes whether or not orthologous genes exist in mouse (Fig. 6b). In particular, we now show human disease genes that are expressed in transgenic mice when the mouse ortholog of that gene does not exist.

#### Expression driven by

Previously, the drivers of expressed allele components have been shown for recombinase-expressing lines in MGI. We have now expanded this to all mutations that contain an exogenous expressed sequence (Fig. 6b). These are displayed in the mutation details section of allele and transgene pages. The species of the driver component is shown, as is the driver entity, with a link to the gene in MGI (for mouse driver genes) or at NCBI (for driver genes from other species).

### MP Browser

Phenotype data can also be accessed from MGI's MP Ontology Browser (https://www.informatics.jax.org/vocab/mp_ontology) (Supplementary Fig. 1a). Terms may be searched in the left search box, and an autocomplete mechanism will suggest valid terms and synonyms. Selecting a term will show the MP term detail including the term name and synonyms, definition, the direct parentage of the term in the ontology, the unique ontology ID and any secondary IDs. Links to the Mouse Developmental Anatomy Browser and GXD expression data are shown when a related anatomical term to the phenotype term is available. The term tree view shows the relationship of the term to all other terms in the ontology. In MGI's browser, a link following the term in the tree view shows the number of MGI genes annotated to the term or its descendants in the ontology tree, and the number of annotations to these terms. Clicking the link will give a detailed list of all annotations, the genotypes annotated and supporting evidence.

### Strains

**Fig. 7.**
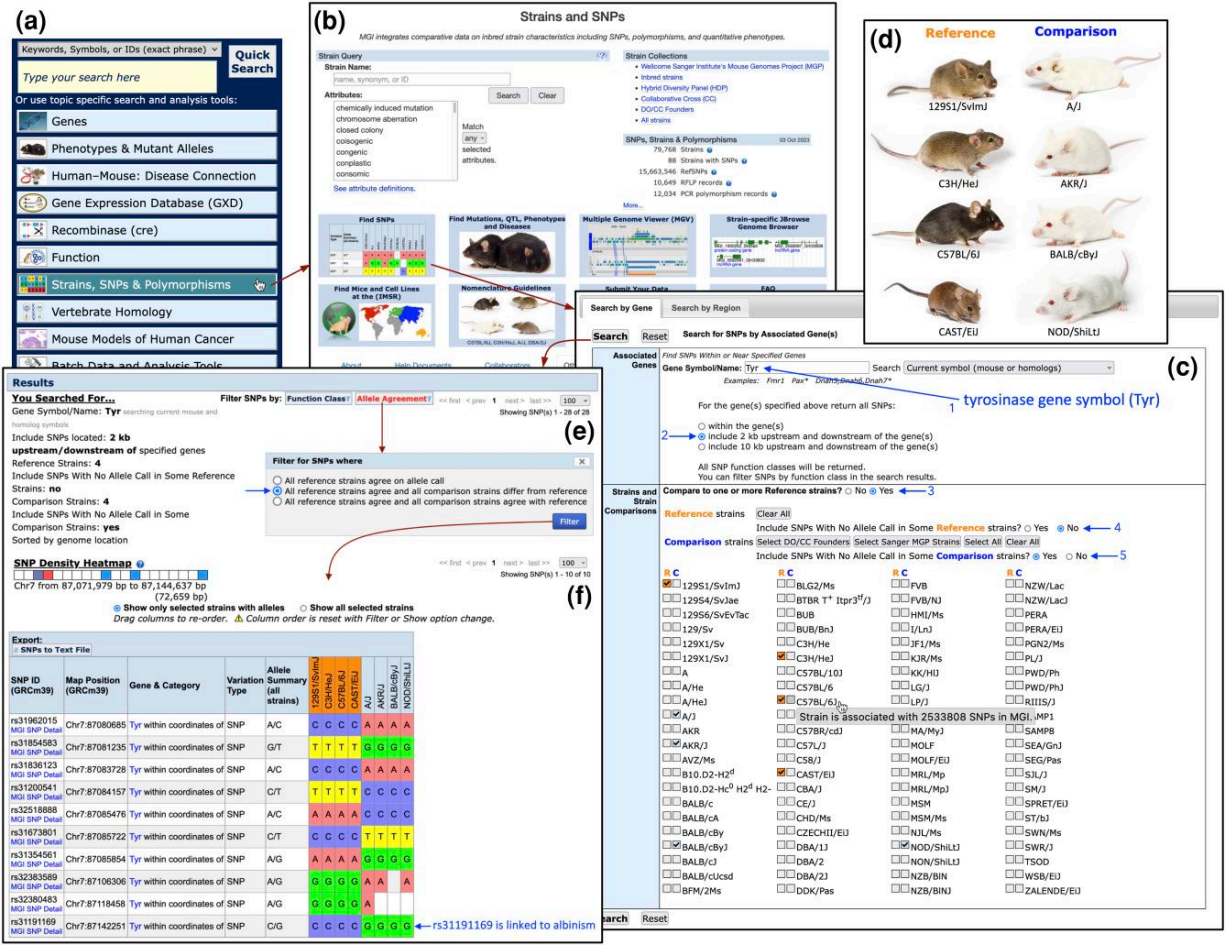
Connecting phenotypes and genotypes with the MGI SNP Query Form. A common path to the SNP Query Form starts by selecting the Strains, SNPs & Polymorphisms option on the MGI Home Page a), which leads to the Strains and SNPs landing page b), which includes the Strain Query form and various strain-related links. Selecting the Find SNPs option leads to the SNP Query Form, with the Search by Gene tab open by default c). The SNP search by gene begins by entering the gene or genes of interest into the Associated Genes section (1st arrow). Gene-based queries provide the option to extend the SNP search upstream and downstream of the gene(s) entered. The “include 2 kb upstream and downstream of the gene(s)” option is selected (2nd arrow). In the Strains and Strain Comparisons section, the option to Compare with one or more References strains is selected (3rd arrow). This option changes the strain display so users can select any strain as a Reference strain (R) or a Comparison strain (C), and mouseover on each strain opens a tooltip that shows the number of SNPs in MGI that involve that strain (see C57BL/6J in the strain selections area). The search specifies that only SNPs with allele calls in all Reference Strains should be returned (4th arrow), but relaxes this constraint for Comparison strains, allowing SNPs with no allele call in some Comparison strains to be returned (5th arrow). All SNPs returned must have an allele call in at least one Comparison strain with this option. The strain selection area lists available strains alphabetically, and when in comparison mode, displays Reference strain (R) and Comparison strain (C) options for each strain. Any number of strains can be selected as Reference or Comparison strains, but a selected strain can only be one or the other. To search for SNPs related to the albino phenotype associated with the tyrosinase gene, the *Tyr* gene symbol was entered in the Gene Symbol/Name field and Reference and Comparison strains were selected based on their coat color. Strains with black or agouti coat color were designated as Reference strains, while strains with albino (white) coat color were the Comparison strains d) (strain images from Jax Mice). The unfiltered search e) returns 28 SNPs (SNPs not shown). Opening the Allele Agreement filter shows three options, the second of which (arrow) restricts results to SNPs for which the allele in all Reference strains is the same AND the allele in all comparison strains differs from the reference strain allele. Applying this Allele Agreement filter restricts results to 10 SNPs f). An SNP density heatmap (panel f) provides an overview of the distribution of SNP results across the input genome region (the Tyr genomic region in this case). The result table lists the SNP rsID, genome location, category(ies) and associate gene(s), type of variation, and the allele summary across all strains. SNPs are sorted by chromosome and genome coordinates. The SNP allele calls for each strain follow, with Reference strains (orange) grouped together and listed before Comparison strains. Two SNPs have no allele call in some comparison strains, a condition allowed in the search parameters (panel c). The last SNP in the result table (rs31191169) has been linked to albinism (Jackson and Bennett 1990; Yokoyama *et al.* 1990; Munz *et al.* 2021).

MGI curates data about mouse strains and stocks. MGI serves as a registry for mouse strains worldwide, maintaining the authoritative nomenclature for existing strains. Comparative data on inbred strain characteristics, SNPs, polymorphisms, and quantitative phenotypes are integrated with other genetic, genomic, and biological data in MGI. Over 79,000 mouse strains are currently listed in MGI and include inbred, mutant congenic and co-isogenic strains, Collaborative Cross strains (Bogue *et al.* 2015), recombinant inbred and other mouse strain types. The Strain and SNPs home page (Fig. 7b) includes a search form for mouse strains by nomenclature or by strain attribute, quick links to lists of strain collections such as the Collaborative Cross, and links to tools and strain resources.

### Strain detail pages

**Fig. 8.**
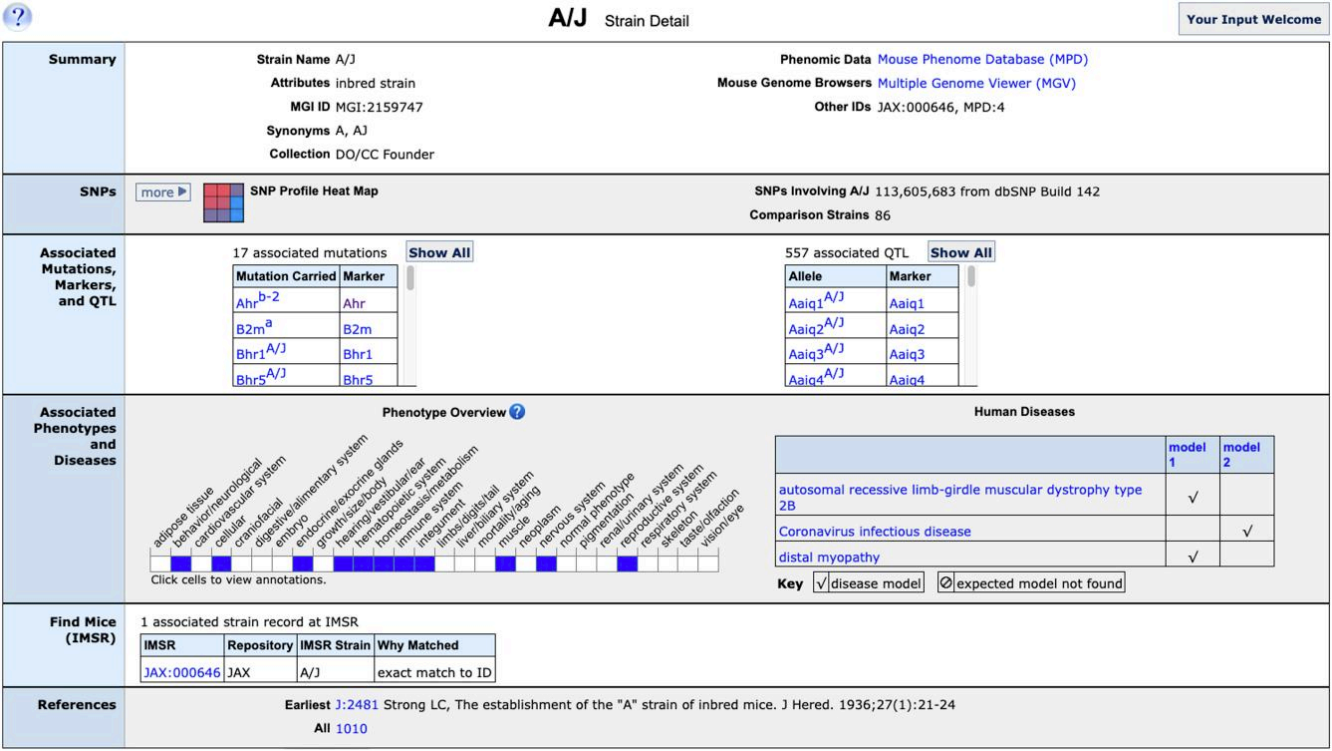
MGI strain detail page for A/J mouse strain. The strain detail page for the inbred mouse strain A/J contains summary at a glance information about the strain. Links are provided to additional strain measurement data at Mouse Phenome Database and to comparative genomes at using the MGV tool. Associated SNP, mutations and QTL, phenotypes and disease are shown and clicking links will show mutation and annotation details. Strain availability is shown via links to IMSR.

Information provided on the strain detail pages shows standardized nomenclature, unique identifiers, repository stock IDs and synonyms (Fig. 8). For many inbred strains, an SNP profile heat map and links to detailed SNP data are provided (see below). Links to curated associated mutation records carried by the strain at MGI, and QTL associated with inbred strains are shown in tabular format. Expertly curated disease model and qualitative strain phenotype characteristics are summarized at a glance in tabular and ribbon display formats with more details available by clicking boxes in the ribbon. Links are provided near the bottom of the strain detail pages to the IMSR (Eppig *et al.* 2015) to provide repository information and availability status at resources worldwide. Finally, selected curated references that show strain characteristics are listed.

### Strain variation resources in MGI

Interstrain genomic variation is fundamental to using mouse as a model to help understand how variation in human populations leads to increased disease risk. MGI offers several strain comparison resources to identify differences between strain genomes at the gene and genome levels.

#### Strain-specific genome features

Although genome features in MGI are assigned genome coordinates in the reference C57BL/6J genome, the features themselves are abstract, canonical representations of the corresponding features present in any species or strain of the *Mus* genus (Richardson *et al.* 2022). Annotations associated with a genome feature on the gene detail page, for example, are not limited to just strain C57BL/6J. By contrast, strain-specific genome features in MGI represent the alleles of a canonical genome feature found in specific mouse strains. MGI has integrated the annotated genome features from 17 fully sequenced mouse strains in addition to the reference C57BL/6J strain, and these annotations can be found for any given genome feature in the Strain Comparison section of the gene detail page (Supplementary Fig. 4). Coordinates for the C57BL/6J strain gene model in the strain comparison table can differ slightly from the coordinates for the canonical gene, since the C57BL/6J strain gene model is derived from the outermost boundary coordinates of the union of gene model annotations from Ensembl and NCBI for that gene. Any of the strains in the table can be selected to obtain corresponding strain-specific FASTA sequences or to open those strain genomes in the MGV, aligned on the denoted genome feature. The Stain Comparison section of the gene detail page includes additional resources designed to facilitate the search for interstrain differences in and around the gene of interest. A count of SNPs within (or up to 2 kb away from) the gene is provided, which links to a summary of these SNPs with allele calls per strain, and links to legacy PCR and RFLP variant data are also provided (Supplementary Fig. 4). Finally, if a genome feature is known to have strain-specific differences a Strain-Specific Marker note is displayed in the Strain Comparison section that describes how the strains differ and with references (if available). The *Ren2* gene (MGI:97899), for example, is absent from the C57BL/6J genome, and the canonical *Ren2* gene detail page displays a Strain-Specific Marker note listing other known strains that lack *Ren2* and some strains known to have the gene. The usefulness of the MGI canonical genome feature model is illustrated by the *Ren2* example.

### SNPs in MGI

MGI has over 15.6 million SNPs involving 88 mouse strains. SNPs in MGI are from dbSNP Build 142, the last dbSNP build with mouse SNP updates. Genome coordinates for all MGI SNPs were updated to GRCm39 using NCBI's Remap tool, and SNP associations to genes, pseudogenes, and other genome features (such as regulatory regions) were established by genome coordinate overlap (see *Methods*). Over 600 thousand genome features in MGI have SNP associations. SNP associations for each genome feature can be viewed from the Strain Comparison section on feature detail pages. There are 393 SNPs associated with the *Pten* gene, for example (Fig. 3). These include SNPs within the gene and within 2 kb upstream or downstream of the gene. SNP summaries are also available for mouse strains from corresponding strain detail pages. The SNPs section on strain detail pages (Fig. 8) includes a strain-by-strain SNP Profile heat map for the featured strain that has links to whole chromosome SNP summaries between selected strain pairs (not shown). Users have several options to search for SNPs in MGI. Searches by SNP ID (rsID) are supported by the Quick Search and the MGI Batch Query tools. More advanced, integrated SNP search options are provided on the SNP Query Form (Fig. 7).

#### MGI SNP Query Form

The SNP Query Form (https://www.informatics.jax.org/snp) is a research tool designed to facilitate SNP searches by strain comparison. The form's strain comparison devices can help narrow the connections between strain phenotypes and causative variant candidates. Users can search for SNPs associated with specific genes (Search by Gene mode, the default) (Fig. 7c) or for SNPs within either a defined region of the genome or within a marker range (Search by Region mode) (not shown). An example of how the SNP Query Form can be used to find SNPs associated with specific phenotypes is shown for a coat color phenotype in Fig. 7. The mouse tyrosinase gene (*Tyr*) encodes a monophenol oxidase (EC 1.14.18.1) that catalyzes the rate-limiting step in melanin production (Seruggia *et al.* 2021). Mice with mutations in the *Tyr* gene have hypopigmentation phenotypes including albinism (reviewed in Seruggia *et al.* 2021). Several inbred mouse strains carry mutations in the *Tyr* gene and have white coat color phenotypes. The Strains and Strain Comparisons section of the SNP Query Form allows specification of one or more Reference strains (R) and Comparison strains (C), and provides two options to widen or concentrate the search, depending on annotation completeness among Reference or Comparison strains (allowing SNPs with no allele calls in some strains, see Fig. 7c). The designation of Reference and Comparison strains is how phenotypes are connected to SNP searches, as these strain groups serve as proxies for the phenotypes being contrasted. By choosing the Reference and Comparison stain sets according to shared phenotypes (Fig. 7c and d), the search can return SNPs that have contrasting allele calls (genotypes) between the strain groups. This is accomplished using an Allele Agreement filter option that restricts results to SNPs that have the same allele call in all Reference strains, and different calls in all comparison strains (Fig. 7e). Applying the Allele Agreement filter option in this example reduces the SNPs returned to 10, one of which (rs31191169) has been linked to the albinism phenotype of the *Tyr^c^* allele (Jackson and Bennett 1990; Yokoyama *et al.* 1990; Munz *et al.* 2021) (Fig. 7f). The *Tyr* gene is associated with 668 SNPs in MGI. The MGI SNP Query Form narrowed this set to a handful of variants where strains that share a phenotype also share a genotype, and at least one of those variants is known to be causative for the phenotype.

## Gene function

MGI's GO project provides functional annotations for mouse gene products using the GO. MGD is one of the founding members of the Gene Ontology Consortium (GOC) (Gene Ontology Consortium *et al.* 2023) and provides major contributions to the development of the GO ontology itself, to common curation software (NOCTUA) development and to developing GO community standards for curation of the scientific literature. Curators at MGI are responsible for annotating mouse genes and gene products to GO ontology terms as well as maintenance of these mouse GO data. Mouse GO annotations are curated by MGI curators using the NOCTUA annotation tool (http://noctua.geneontology.org/workbench/noctua-landing-page/) at the central GOC resource. In addition, mouse GO annotations from other resources, primarily the UniProt-Gene Ontology Annotation project (HUNTLEY et al. 2015), are integrated at GOC and imported to MGI. MGI GO data currently includes 524,089 annotations to 30,702 protein-coding and nonprotein coding genes (Table 5).

**Table 5.** Gene Ontology data in MGI.

Genes with GO annotations	30,702
Genes with experimentally derived GO annotations	12,643
Total mouse GO annotations	524,089

Numbers of Gene Ontology annotations to genes in MGI. Gene counts include protein-coding and noncoding RNA genes. Gene counts shown from experimentally derived annotations only consider direct annotations from experiments in mice and exclude annotations inferred by sequence similarity and other inferred methods.

### Gene ontology (GO) classifications

The GO Classifications section of the gene detail page shows a summary view of these annotations (Fig. 3). A link to a functional annotation detail page is provided and shows the number of mouse annotations available to view. A link to a summary listing of references containing gene function data for mouse is shown with the number of references available. A high-level summary view ribbon of these annotations is shown and includes terms related to molecular function, biological process and cellular component. A blue square indicates that data is annotated for that high-level term or any of its descendants in the ontology. Clicking on the square will show a page with the annotation details for the term selected and any descendants of that term in the GO ontology.

**Fig. 9.**
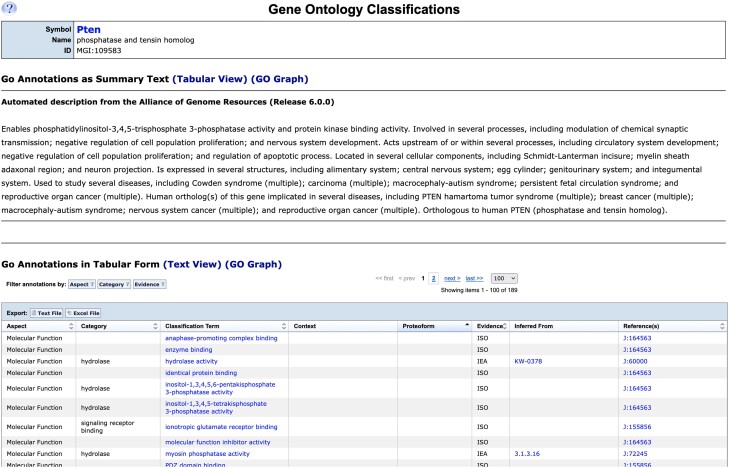
MGI Gene Ontology classifications for the mouse *Pten* gene. The detail page for all GO annotations to the mouse *Pten* gene found when clicking the “all” annotations link from the gene detail page is shown. The summary functional information provided by the Alliance of Genome Resources is shown at the top of the page and a tabular summary of annotations is shown at the bottom. A graphical view is also available (not shown).

Mouse GO annotations are loaded weekly from GOC to MGI and are displayed on the Gene Ontology Classifications detail page (Fig. 9). The page shows an automated gene summary description paragraph (Alliance of Genome Resources Consortium 2020) imported from the Alliance of Genome Resources and includes major information about the function, phenotypes and diseases related to the gene. GO annotations are also shown in a tabular format, listing each annotation separately. Each annotation is shown with supporting evidence, context, and references. Results can be filtered and downloaded in text or Excel format.

### GO Browser

GO data can also be accessed from MGI's Gene Ontology Browser (https://www.informatics.jax.org/vocab/gene_ontology) (Supplementary Fig. 1b). The three aspects of the GO ontology (Molecular Function, Cellular Component, and Biological Process) can be viewed separately by clicking on the links at the top of the browser window. Terms may be searched in the left search box, and an autocomplete mechanism will suggest valid terms and synonyms. Selecting a term will show the GO term detail including the term name and synonyms, definition, any comments, the direct parentage of the term in the ontology, the unique ontology ID and any secondary IDs. The GO tree view shows the relationship of the term to all other terms in the ontology. In MGI's browser, a link following the term in the tree view shows the number of MGI genes annotated to the term or its descendants in the ontology tree, and the number of annotations to these terms. Clicking the link will give a detailed list of all annotations, the genes annotated with the annotations and supporting evidence.

### Protein information

MGI incorporates several types of protein data from external sources and summarizes this information for each protein-coding gene in the Protein Information section of the gene detail page. In the Protein Information section of mouse *Pten*, for example (see Fig. 3), the PTEN Protein Ontology (PRO) classification (Natale *et al.* 2017) is shown with links to the corresponding PRO Report and term hierarchy. Enzyme Commission (EC) numbers (Bairoch 2000) and InterPro domains (Paysan-Lafosse *et al.* 2023) are also listed, with links to details at resource websites. We also load Protein Data Bank (PDB) associations (Berman *et al.* 2000) (not shown for *Pten*), and provide links to PDB details in this section for associated genes. We obtain EC, InterPro and PDB associations to mouse genes from UniProt (UniProt Consortium 2023), and include another link to a summary of UniProt sequences for the gene in the Protein Information section.

## Gene expression

The mouse Gene Expression Database (GXD) provides the expression information in MGI. GXD's focus is on endogenous gene expression in wild-type and mutant mice. Different types of expression data at the RNA and protein level are collected to furnish detailed information about expression profiles. The expression data are fully integrated with the genetic, functional, phenotypic, and disease related information collected by MGD, thus facilitating insights into the molecular mechanism of health and disease (Ringwald *et al.* 2022).

### Classical types of expression data

For many years, GXD has collected mouse developmental expression data from RNA in situ hybridization, immunohistochemistry, in situ reporter (knock in), RT-PCR, northern blot, and western blot experiments. These data are acquired through systematic curation of the scientific literature and by collaborations with large-sale expression projects (Finger *et al.* 2017; Smith *et al.* 2019).

#### Gene Expression Literature Index

As a first step in our literature annotation work, GXD curators identify all new publications documenting endogenous gene expression during mouse development, and they index these publications with regard to the genes that have been studied, the expression assay types used, and the ages analyzed. These annotations are combined with bibliographic information from PubMed to generate the Gene Expression Literature Index (Smith *et al.* 2014). The index is complete and up to date. It covers all relevant articles published from 1993 to the present, and all articles published in major developmental journals from 1990 to the present. As of 2023 November 27, the Gene Expression Literature Index contains 272,537 entries covering 32,842 references and containing expression information for 16,951 genes. Thirty-four percent of the reported assays are RNA in situ hybridization experiments, followed by Immunohistochemistry (31%) and RT-PCR assays (23%), reflecting the spatial resolution and sensitivity required for developmental expression studies. All literature index information is readily searchable via the Gene Expression Literature Search (https://www.informatics.jax.org/gxdlit). This search form is a highly effective tool to rapidly find publications with specific types of expression information. It is more effective and complete than PubMed searches because the index uses standard nomenclature for genes, assay types, and ages, and because the annotations are based on the entire article, including Supplementary Material. The Gene Expression Literature Index also provides the basis for our work on the detailed annotating of expression data from the literature and for prioritizing this task.

#### Annotation of detailed expression data

**Fig. 10.**
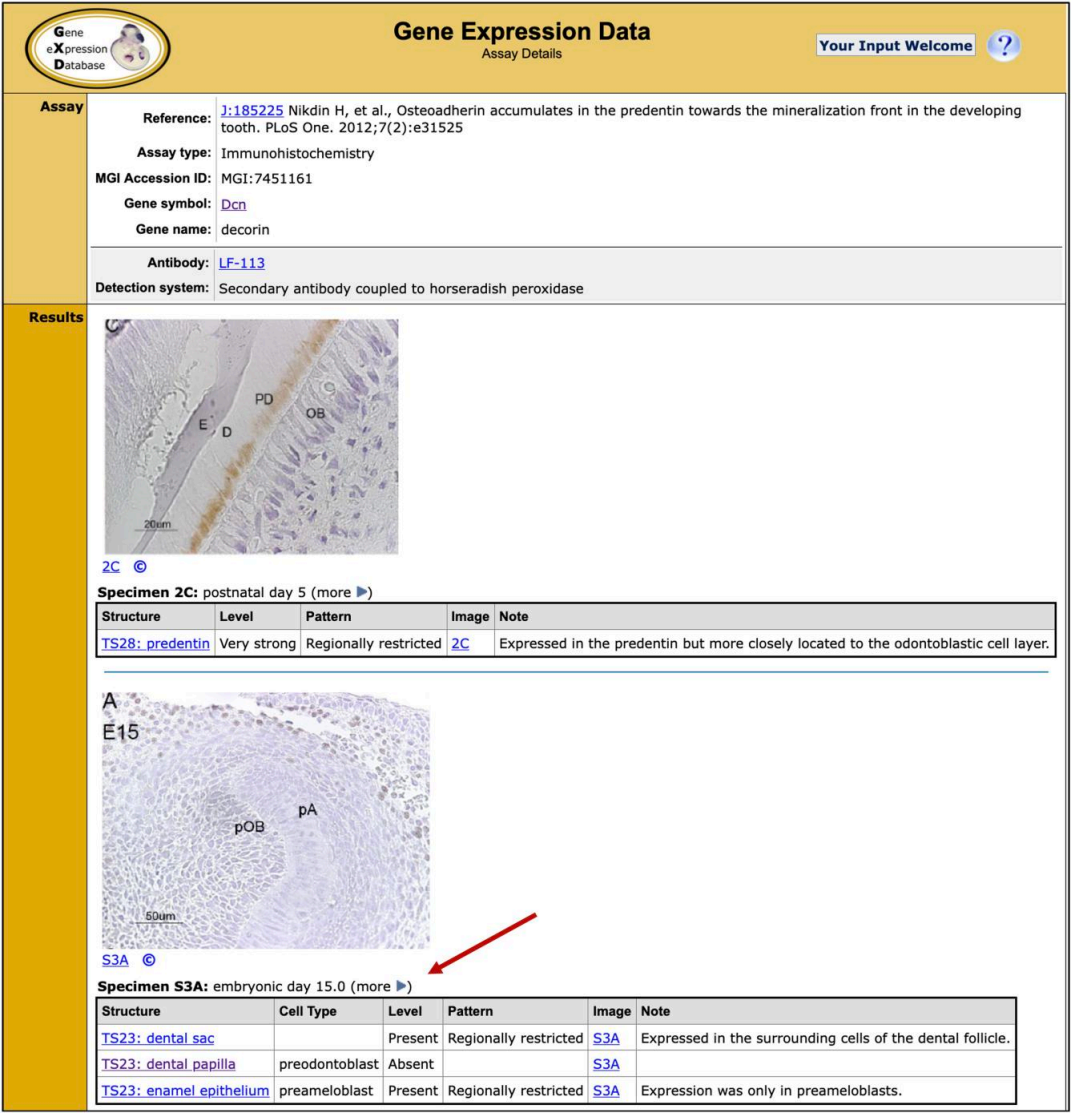
Assay detail page for an immunohistochemistry experiment. Only the upper part of the entry is shown. Arrow points to the expression results for specimen S3A, which are annotated in a modular way by combining terms from the anatomy and cell ontology.

A major objective of our data acquisition work is to annotate detailed expression data from articles identified through our GXD Indexing work, from electronic data submissions, and from consortia that generate this type of expression data on a large scale. Consortia and project-specific databases from which we have integrated large-scale in situ data include GenePaint (Visel *et al.* 2004), Eurexpress (Diez-Roux *et al.* 2011), BGEM (Magdaleno *et al.* 2006), GUDMAP (Harding *et al.* 2011), and the IMPC (Koscielny *et al.* 2014). We bring all these data into standardized formats and integrate them with other information in GXD and MGI. The annotation work makes extensive use of controlled vocabularies and ontologies. A detailed entry is shown in Fig. 10. As of 2023 November 27, GXD contains detailed and standardized annotations for 133,635 expression assays that provide 1,968,735 expression results for 15,944 genes. Expression records are linked to digitized images of the original expression data. Currently, GXD includes 466,814 images. All these images are accessible via many different queries (see below) due to the standardized metadata that our annotations provide. A large proportion of the data are from RNA in situ hybridization and immunohistochemistry experiments, many of which include data from mutant mice, illustrating the biological complexity of the expression data captured by GXD. At present, GXD holds expression data from 7,849 different mutants. See Table 6 for additional information on GXD's current data content. GXD uses the Mouse Developmental Anatomy (EMAPA) Ontology to annotate the time and space of gene expression. Initially developed in collaboration with the Edinburgh Mouse Atlas Project (EMAP), GXD continues to maintain and extend this ontology (Hayamizu *et al.* 2015). It currently includes over 34,000 stage-specific anatomy terms. However, because it is unfeasible to extend this representation down to the cellular level due to the large number of required hierarchical levels and leaf nodes, we recently began to include the Cell Ontology (CL) (Diehl *et al.* 2016) as an additional standardized descriptor of expression patterns. As illustrated in Fig. 10, publications or electronic submissions reporting expression in a particular cell type and tissue are now annotated in a modular fashion by using terms from the anatomy and cell ontology. This will enable additional search capabilities, as well as further integration with scRNA-Seq data.

**Table 6.** Gene expression data in MGI.

GXD assay type	Expression results	Genes	Anatomy terms	Mutant alleles	Images
RNA in situ	1,358,788	13,214	13,954	1,828	274,852
RT-PCR	222,528	9,316	2,479	2,081	16,509
Immunohistochemistry	154,836	3,673	10,944	3,215	69,219
In situ reporter (knock in)	133,750	3,282	6,531	3,930	98,538
Northern blot	52,591	3,377	960	549	2,915
Western blot	46,242	3,017	1,136	1,567	4,781
Classical Expression totals	1,968,735	15,944	17,217	7,849	466,814
RNA-Seq	36,724,708	52,018	178	120	NA

Total numbers of GXD expression results (column 2) are shown distributed by assay type. Except for high-throughput RNA-Seq data (last row), all data are curated from published literature or from contributed datasets. The numbers of associated genes, stage-specific anatomical structures (EMAPS terms), mutant alleles, and images are also shown for each assay type. Counts include positive and negative expression results in the structures assayed. Classical gene expression assay types separated from RNA-Seq. Total distinct counts for all classical gene expression assay types are shown in the second to last row. RNA-Seq results represent average quantile-normalized TPM values for each gene from combined technical and biological replicate samples (Baldarelli *et al.* 2021) (image counts not applicable for RNA-Seq experiments). Classical types of expression data are currently annotated to over 17,000 distinct stage-specific anatomical structures whereas the RNA-Seq experiments include 178 distinct anatomical structures. This nicely illustrates the complementary value of classical and RNA-Seq experiments. While RNA-Seq experiments cover the entire genome and generate large volumes of expression results per experiment, classical types of expression experiments, in particular RNA in situ hybridization and immunohistochemistry, yield more detailed spatial expression information.

### RNA-Seq and microarray expression data

In recent years, we extended GXD to include RNA-Seq and microarray expression experiments. As with classical types of expression data, our focus remains on endogenous gene expression in wild-type and mutant mice.

#### RNA-Seq and microarray experiment index

It is difficult to find specific RNA-Seq and microarray experiments in GEO (Clough and Barrett 2016) or ArrayExpress (Athar *et al.* 2019) using biological search parameters because sample annotations are limited and often rely on free-form metadata provided by data submitters. To address this issue, we set out to annotate metadata for high-throughput expression datasets from ArrayExpress and GEO using the detailed controlled vocabularies and ontologies employed in GXD/MGI. Because ArrayExpress used to import RNA-Seq and microarray experiments from GEO, we initially identified all RNA-Seq and microarray experiments in ArrayExpress that were within GXD's scope; annotated the metadata for GXD-relevant datasets by attributes of the samples used (e.g. strain, tissue, age, sex, mutation carried) as well as by experimental categories (baseline tissue comparison, wild-type vs mutant, etc.); and created a standardized, searchable index of RNA-Seq expression and microarray experiments in ArrayExpress and GEO to help researchers quickly and reliably find datasets of interest (Smith *et al.* 2020). Because ArrayExpress stopped incorporating datasets from GEO, we implemented an additional pipeline for importing and annotating data directly from GEO; and we began to identify and curate GXD-relevant datasets in GEO that are not available in ArrayExpress. We also added cell type as an additional standardized descriptor for indexing RNA-Seq and microarray experiments in GEO and ArrayExpress. As of 2023 November 27, we have identified 6,614 experiments as GXD-relevant experiments based on curatorial review, and have annotated 6,083 of these experiments. The searchable index is available via the RNA-Seq and Microarray Experiment Search (https://www.informatics.jax.org/gxd/htexp_index).

#### Curation and integration of RNA-Seq expression data

The EBI Expression Atlas project selects high-quality RNA-Seq datasets from ArrayExpress and GEO and uses a standardized processing pipeline, starting from the primary data, to generate consistently processed TPM values (Papatheodorou *et al.* 2018). We have imported these TPM level data from the Expression Atlas for those RNA-Seq experiments that are within GXD's scope. By taking advantage of our sample metadata annotations (described above), we have processed these data further to enable their full integration with classical types of expression data in GXD thus making them accessible through the search, filtering, and display tools that GXD provides (Baldarelli *et al.* 2021). As of November 2023, we have integrated expression data for 96 RNA-Seq experiments. These include 3,309 distinct samples, representing 178 distinct anatomical structures and 106 distinct mouse strains. Mouse genes represented in Expression Atlas RNA-Seq TPM files cover the entire Ensemble transcriptome (over 52,000 genome features), including protein-coding and noncoding RNA genes. Total RNA-Seq assay results amount to nearly 37 million, with comprehensive genome coverage for each experiment. See also Table 6.

### Search and display features

GXD's interface utilities have been described previously (Finger *et al.* 2015; Finger *et al.* 2017; Smith *et al.* 2019, 2020; Baldarelli *et al.* 2021). Here, we provide a brief summary and describe novel search features in more detail.

### Search forms

The GXD Home Page (https://www.informatics.jax.org/expression.shtml) provides the best entry point to all the features and resources provided by GXD. Graphical tiles provide a quick overview of, and access to, GXD's search functions:

*Expression Data and Image Search*: Search for expression and image data using many different parameters. This is our most versatile web-based search form.

*Differential Expression Search*: Search for genes expressed in an anatomical structure and/or developmental stages but not in others.

**Fig. 11.**
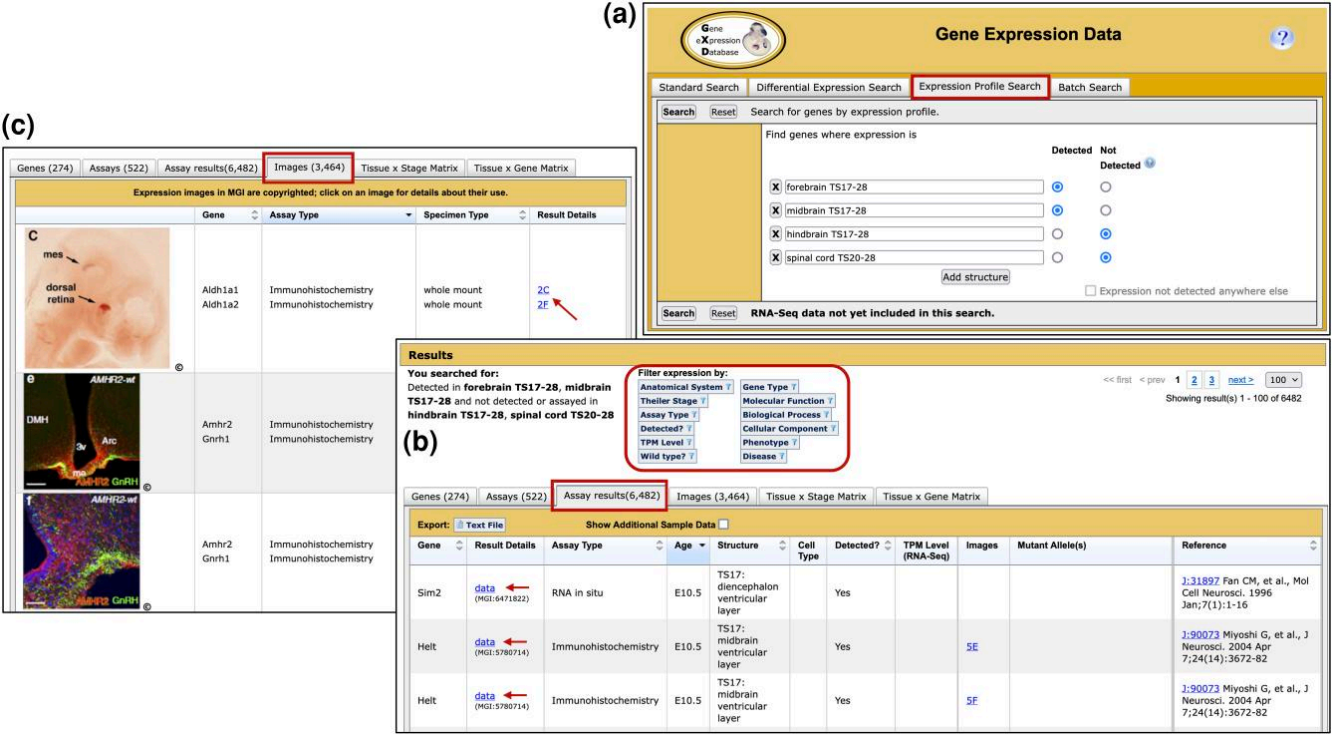
GXD Expression Profile Search. a) Expression Profile query form showing a search for genes with expression detected in two structures (forebrain and midbrain) and not detected (or assayed in) two structures (hindbrain and spinal cord). Conditions for each row of the profile are combined with a Boolean AND in the query. b) Assay results tab of resulting search summary, filtering options are encircled in red. c) Images tab from the same search summary, showing the first 3 of 3,464 images. Arrows in panels b and c indicate links from the summaries to Assay Detail pages (such as the one shown on Fig. 10).

*Expression Profile Search*: This newly added tool allows users to search for genes with an expression profile of interest by specifying up to 10 anatomical structures and whether expression is present or absent in these structures. In addition, users can search for genes that are expressed in a set of anatomical structures and nowhere else. See Fig. 11.

*Search Using Gene List*: Using this newly implemented batch search utility, one can retrieve GXD's expression data for large lists of genes (such as, interesting genes identified in RNA-seg experiments).

*Developmental Anatomy Browser*: Allows users to browse and search for anatomical structures and to look up the expression data and the phenotype data associated with a given anatomical structure.

*Tissue-by-Stage Matrix*: Interactive matrix providing access to all classical types of expression data in GXD. The tissue axis can be expanded and collapsed based on the hierarchical structure of the anatomy ontology.

*Expression Literature Search*: Allows users to quickly find publications using the curated Gene Expression Literature Index described above.

*RNA-Seq and Microarray Experiment Search*: Allows users to quickly find RNA-Seq and microarray experiments of interest, based on GXD's metadata curation (described above).

*MouseMine API Data Access*: Provides easy programmatic access to GXD data.

The Differential Expression Search, Expression Profile Search, and the MouseMine API are currently limited to classical types of expression data. Access to RNA-Seq data will be added in future releases.

### Interactive search summaries and unified data navigation

As shown at www.informatics.jax.org/mgihome/GXD/FirstTimeUsers.shtml, all of GXD's expression data search forms lead to the same multitabbed displays that summarize data at different levels of detail: Genes, Assays, Assay Results, Images, and in the form of two different Matrix Views, Tissue × Stage and Tissue × Gene Matrix. These summaries (two of which are shown in Fig. 11b and c) can be sorted and refined further by many filtering options. For example, one can filter for sets of genes based on their gene type (protein-coding, noncoding RNA, etc.), their phenotype and disease associations, their molecular functions, the biological processes they are involved in, and the cellular components in which they are found, and expression results can be filtered by anatomical system, developmental stage, assay type, detected (yes/no), etc. Filtering actions chosen in one summary view are applied to all summaries so that all views are in sync with regard to their data content. Summaries lead to detailed expression entries, such as the one shown in Fig. 10. Data summaries can also be downloaded and exported to external applications. Notably, by merely clicking a button, one can export search summaries of RNA-Seq data into Morpheus. Morpheus, a heat map visualization and analysis tool developed at the Broad Institute, offers myriad utilities for further display and analysis, including sorting, filtering, hierarchical clustering, nearest neighbor analysis, and visual enrichment. The export function transmits GXD's sample annotations to Morpheus, where they can be readily used for sorting, filtering and clustering (Baldarelli *et al.* 2021).

### Gene-specific expression summaries and links to other expression resources

Users who are just interested in the expression information for a specific gene can find access to all the relevant information in the expression section of the corresponding gene detail page. As shown in Fig. 3 for the gene *Pten,* the expression section of gene detail pages summarizes the expression information for a given gene. A graphical ribbon provides a high-level overview of gene expression in specific anatomical systems with links to the corresponding system-specific tissue-by-state matrices. A link to the entire tissue-by-gene expression matrix for the gene is available, as are links to the gene-specific assay results and image summaries. The Literature Summary lists all publications for the gene in GXD's Gene Expression Literature Index. In addition, the expression section of the gene detail page provides gene-based links to other mouse expression resources such as the Allen Institute (Sunkin *et al.* 2013), GENSAT (Heintz 2004), NCBI-GEO, and the EBI Expression Atlas, as well as links to gene expression information in other MODs highly relevant for developmental research, i.e. to GEISHA (chicken) (Antin *et al.* 2014), Xenbase (Xenopus) (Fortriede *et al.* 2020), and ZFIN (zebrafish) (Bradford *et al.* 2022). To further support cross-species expression analysis, GXD also provides its data to the Alliance of Genome Resources (Alliance of Genome Resources Consortium 2022). All mouse expression data available from the Alliance of Genome Resources come from GXD.

### Anatomical comparison of expression and phenotype data

**Fig. 12.**
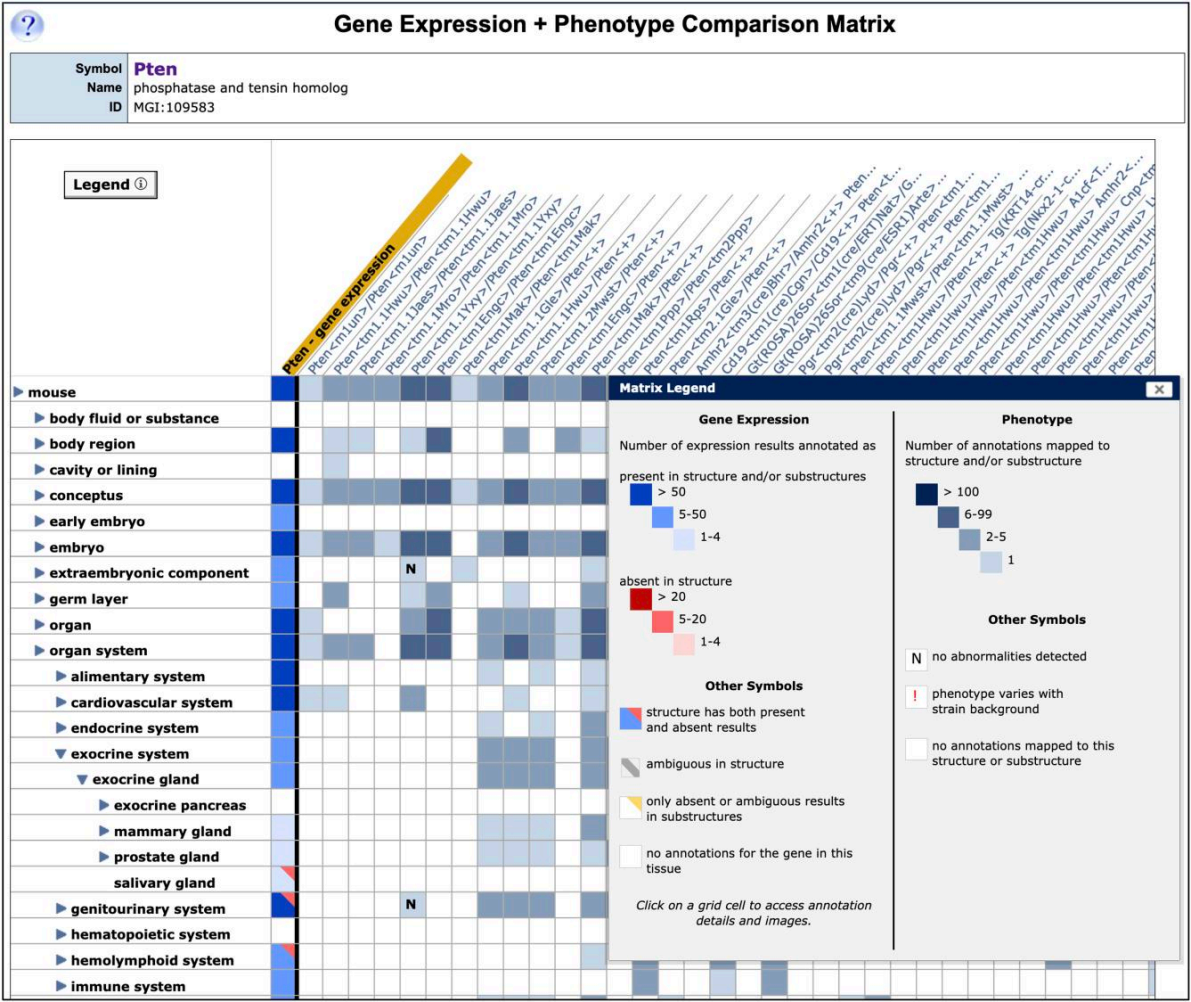
Comparing the expression and phenotype pattern for a specific gene. The Gene Expression + Phenotype Matrix displays the expression and phenotype data for a selected gene in the same anatomical matrix view. The gene *Pten* is shown as an example, with the “exocrine system” expanded along the anatomy axis. The wild-type expression pattern of *Pten* is displayed in the first column (gold header). The following columns show the anatomical structures phenotypically affected in different *Pten* mutant mice (different *Pten* alleles). The coloring of the matrix cells gets progressively darker as the number of expression and phenotype annotations increases; the conventions are defined in the matrix legend (inset).

Anatomical ontologies are important integration hubs for biomedical data because many types of biological data relate to specific anatomical structures and systems. To enable the anatomical comparison of expression and phenotype data, we have recently mapped terms from the MP Ontology to terms in the Mouse Developmental Anatomy (EMAPA) Ontology. Due to these mappings, users can now look up the expression data and the phenotype data associated with a given anatomical structure, using the Developmental Anatomy Browser, as described above. In addition, we have developed the Gene Expression + Phenotype Comparison Matrix. This interactive matrix displays both the expression and phenotype data in the mouse developmental anatomy framework. It visually juxtaposes the tissues where a gene is normally expressed against tissues where mutations in that gene cause abnormalities, and thus allows researchers to explore if there is a correlation between gene expression and phenotype for a given gene (Fig. 12). The Gene Expression + Phenotype Comparison Matrix is accessible from the expression and phenotype sections on MGI gene detail pages, see Fig. 3.

## Human diseases

### Summary human disease data on the gene detail page

The Human Diseases section of the gene detail page shows a summary of MGI data that lists known human diseases associated with the gene, and if mouse models have been identified for that human disease (Fig. 3). Opening the data toggle will reveal a summary table (Supplementary Fig. 5), which is divided into three sections: disease associations with the gene in both human and mouse (both icons), curated disease associations with only the mouse gene (mouse icon), and disease associations with only the human gene (human icon). Clicking on the disease name will open a disease detail page (see *Disease Ontology Browser* section) that will list all genes and models associated with that gene. Clicking on the ID link will show all mapped IDs from multiple resources and links to several of these (OMIM, ORDO, MESH, DOID, etc.) provide more information about each of these human diseases. The Mouse Models column lists the number of curated mouse models for each disease (if present) and the link opens a popup that lists the individual models with further links to detailed information about these models.

### Disease Ontology Browser

The DO project has been developed as a standardized ontology for human disease and contains descriptions of human disease terms, phenotype characteristics and related medical vocabulary disease concepts (Schriml *et al.* 2019). MGI uses the DO to annotate models of human disease. Annotated data can be accessed through MGI's DO Browser (https://www.informatics.jax.org/disease) (Supplementary Fig. 1c). Detail pages include the term and unique ID, a link to the Alliance disease ontology page for comparative genomics in other species, synonyms, other IDs and the text-based definition. The term browser tab includes parent and child terms in the ontology structure. The genes tab includes human genes and mouse genes, transgenes and other complex mutations annotated to the disease. Further links are provided if a mouse model of that gene or mutation is annotated at MGI. The Models tab lists all mouse genotypes annotated as a mouse model of the human disease, plus supporting references and a further link to detailed phenotype data for that genotype. Finally, a link to a list of selected references that discuss mouse models of the featured disease is shown at the bottom of the page.

### Access to human disease data via the HP Ontology Browser pages

The HP Ontology Project provides a standardized vocabulary of phenotypic abnormalities encountered in human disease and annotates these terms to human diseases (Gargano *et al.* 2023). These data have been incorporated into MGI and can be accessed from MGI's HP Ontology Browser (https://www.informatics.jax.org/vocab/hp_ontology) (Supplementary Fig. 1d). Terms may be searched in the left search box, and an autocomplete mechanism will suggest valid terms and synonyms. Selecting a term will show the MP term detail including the term name and synonyms, definition, the direct parentage of the term in the ontology, the unique ontology ID and any secondary IDs. The term tree view shows the relationship of the term to all other terms in the ontology. In MGI's browser, a link following the term in the tree view shows the number of human diseases associated with the phenotype term or its descendants in the ontology tree, and the number of annotations to these disease terms. Clicking the link will give a detailed list in the HMDC (see below) of all the human diseases associated with the phenotype term, the human and mouse genes associated with the diseases and supporting evidence.

### Human–Mouse: Disease Connection

To facilitate cross-species translation of phenotype and disease data between human and mouse we have developed the HMDC tool. This tool allows for users to pose simple to complex questions and retrieve phenotype and disease data for both human and mouse genes simultaneously. Human data are imported from the HP Ontology knowledgebase. Human genes are associated with HP ontology terms and human diseases from the Online Mendelian Inheritance in Man (OMIM) and Orphanet resources. Mouse genes are associated with MP Ontology terms and Human DO terms based on annotations made by MGI. Cross-references in the DO allow for most of the OMIM and Orphanet diseases to be associated with DO terms.

**Fig. 13.**
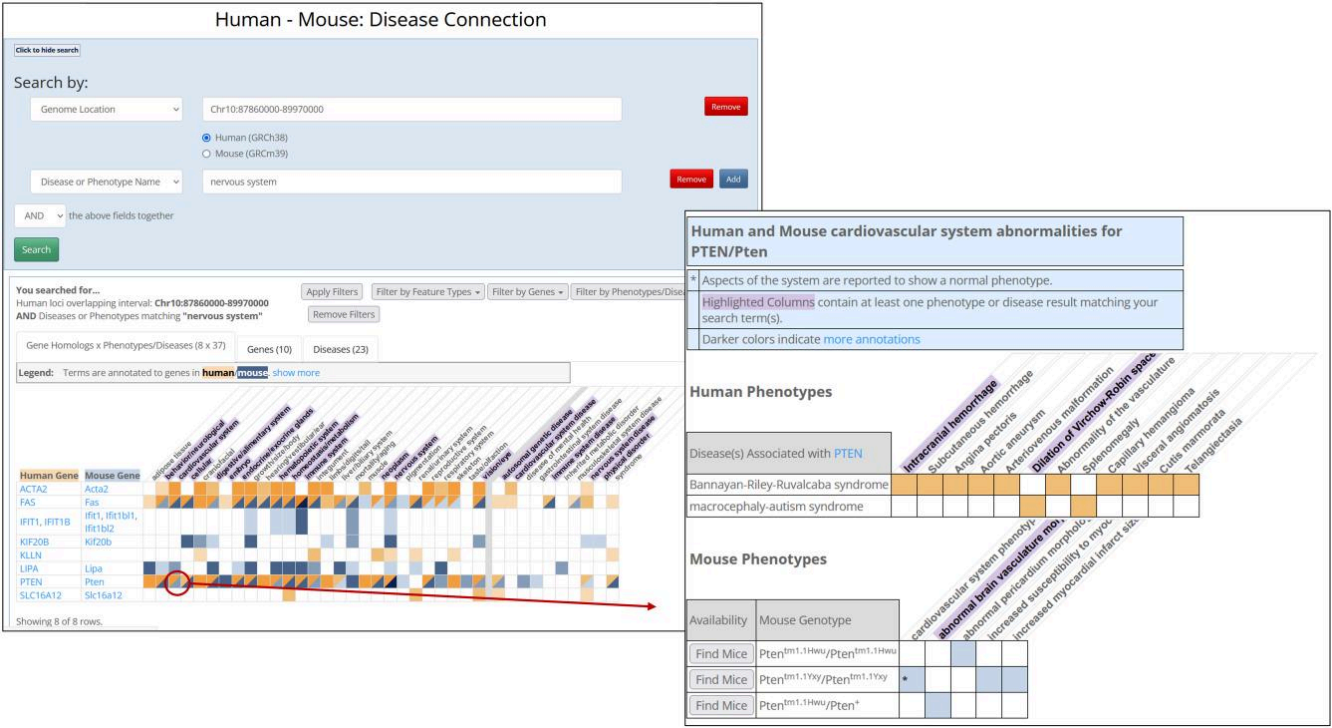
The human–mouse disease connection search and results. a) The upper section of the HMDC search tool allows you to select multiple fields to search using either Boolean AND or OR. Shown are two selected search fields, Human Genome Location and Disease or Phenotype Name. The chromosome 10 region spanning from 87860000 to 89970000 is entered in the search box and human was selected from the radial buttons below the search box. Searching by a human region returns all the human genes included or overlapping that region and all of the mouse orthologs of those human genes. A second search field was selected by using the “Add” button and selecting Disease or Phenotype Name. To search for genes associated with nervous system phenotypes “nervous system” was entered in the search field. Name searches will look for terms in the MP, HP, or DO that contain all of the words entered in the text field. Annotations to the matching term or any descendent of the matching term are included in the results. Thus, the search for nervous system will return phenotypes like tonic–clonic seizures (MP:0003997), Meningioma (HP:0002858), or familial meningioma (DOID:4586) as they are descendants of terms that include the words “nervous system” in each the ontology. b)The lower section shows search results organized into three tabs. The first results tab (shown) is a summary overview grid of gene by phenotype and disease. In the example search, the grid includes for human genes all the phenotypes and diseases for any disease that is associated with at least one phenotype in the nervous system. For the mouse genes the grid includes all phenotypes and diseases for genotypes containing alleles of that gene that have at least one nervous system phenotype. For example, the genotype *Pten^tm1.1Hwu^/Pten^tm1.1Hwu^* is included in the results as it has the phenotype “wavy neural tube” and the human ortholog PTEN is in the chromosome region. However, the genotype *Pten^tm1.1Hwu^/Pten^tm1.1Hwu^* is not included in the results set because it does not have any annotations for nervous system phenotypes. Human data are represented by shades of orange in cells of the summary grid, while mouse data are represented by shades of blue. Darker shades indicate more data. Columns that contain at least one matching term in at least one cell are highlighted in purple. In the figure, the column for “immune system” is highlighted because the term “brain inflammation” (MP:0001847) has parents in both the immune and nervous systems. c) Clicking on a cell in the grid will open a popup that displays the detailed annotations. The popup for the cell “cardiovascular system X PTEN/*Pten*” shows the set of HP or MP terms in the cardiovascular system for each disease associated with human PTEN or for each genotype for mouse *Pten*. Clicking on a row with human data will open the term at the HPO website. Clicking on a row with mouse data will open the genotype detail page in MGI.

The HMDC supports searches by gene symbol, gene ID, phenotype name, disease name, phenotype ID, disease ID, or genome region. These various searches can be combined to allow for users to search by multiple criteria at the same time. For example, you can combine a search for the human region Chr10:87860000–89970000 that includes both *PTEN* and *FAS* with the partial phenotype term “nervous system” to limit your results to only genes in that region that are associated with at least one phenotype in the nervous system. While the search criteria specified a human region the search results include both human genes in the region and the mouse orthologs for those genes (Fig. 13). Using a phenotype or disease term name will search across all three ontologies in the HMDC. For example, “nervous system” will match the HP term “Abnormal nervous system morphology” (HP:0012639), the MP term “abnormal nervous system physiology” (MP:0003633), and the DO term “nervous system disease” (DOID:863). The results section is shown in three tabs. The first tab is a summary grid overview of gene by phenotype and disease, and the dataset returned includes genes associated with any of these search terms or their descendants in the ontology used. This casts a broad net to bring in data from both species regardless of the ontology used to capture the data. The second tab (not shown) in the results section lists all genes and summary disease and phenotype data annotated to the search terms presented in a tabular and downloadable format. The third tab (not shown) in the results section lists all human diseases associated with genes matching the search criteria, the human gene and mouse models of the returned diseases, also in a tabular and downloadable format.

**Fig. 14.**
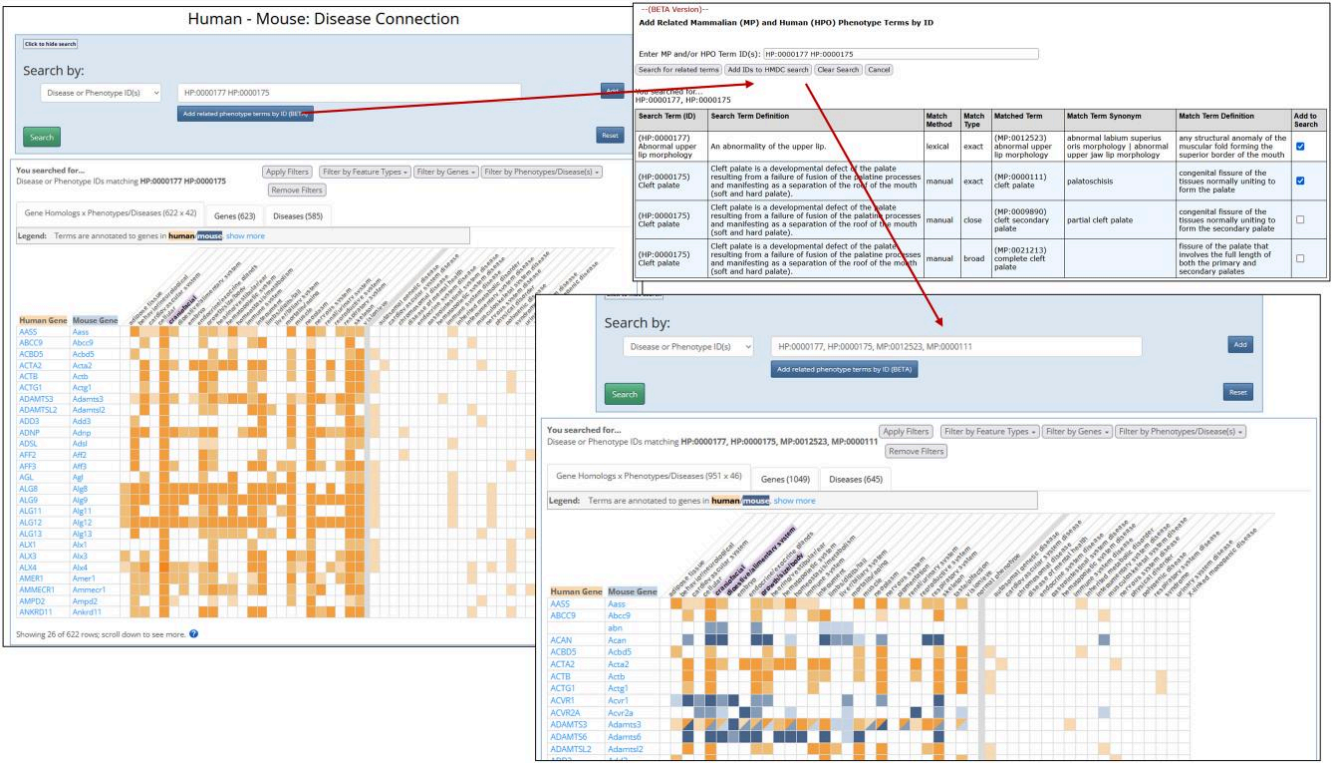
The human–mouse disease connection term matching tool. a) When searching the HMDC by IDs, multiple IDs in a single field are connected by Boolean OR in the search. Searching by HP IDs returns only human genes and searching by MP IDs returns only mouse genes as the ID search does not go across ontologies unlike the name search. In the image searching for “HP:0000177, HP:0000175” returns human genes associated with either Cleft palate (HP:0000175) or Abnormal upper lip morphology (HP:0000177) but no mouse genes as seen by the presence of only orange cells in the grid. Clicking the “Add related phenotype terms by ID (BETA)” button opens the MP–HP matching tool and fills in the search box with any IDs already entered in the HMDC search box. b) Multiple IDs can be searched for at the same time. Sets of matched terms are grouped and zebra striping indicates each grouping. The search term and definition of that term in the respective ontology are shown in the first 2 columns. Match Method and Match Type are shown in the next 2 columns. The matched term, synonyms, and definition are in the next 3 columns. Synonyms and definitions are taken from the respective ontology. The final column allows the user to select specific terms for inclusion in the search. Clicking “Add IDs to HMDC search” adds all selected IDs and any IDs entered in the search box to the HMDC search. c)After adding terms from the match tool and running the search again the grid now includes both human and mouse genes that are associated with at least one of the phenotype terms.

As mouse phenotypes are captured using the MP while human phenotypes are captured using the HP searches using a term ID will return data for only a single species. Searching by IDs allows for searches for multiple phenotypes at the same time. IDs entered in the same search box are connected with the Boolean OR. To help find related terms in each ontology, we have created an MP–HP matching tool (Fig. 14). This tool uses files from the Mouse-Human mapping repository (https://github.com/mapping-commons/mh_mapping_initiative) that contain relations between the MP and HP ontologies. Relations in these files are created using a variety of methods. The method used for matches is shown in the results table in the tool. Match methods include: manual, created by expert curation; lexical, automated matches made by comparing term labels and synonyms; and logical, automated matches made using logical definitions in the ontologies. These files are also supplemented in the tool with additional matches generated by MGI by making lexical matches between term labels and synonyms. The MGI lexical matches for nonexact synonyms use the synonym types to determine the match type. Match types are not limited to only exact matches but also use close, broad, narrow, and related match types to increase the pool of potential terms for the user to add to the search. Match types are defined in the Simple Knowledge Organization System (SKOS) standard (Matentzoglu *et al.* 2022). Searching with MP and/or HP IDs will find any matches made by any method in the opposite ontology. If the same MP-to-HP match is found by multiple match methods, only a single match method will be displayed in the tool, with preference given to more confident match methods (manual > logical > lexical). As the different types of matches may be more or less useful depending on the goal of the user, the results table of the tool allows the user to select the set of terms to include in the HMDC search. To aid in selection, the definitions and synonyms of both terms are included for each mapped pair.

## Future directions

MGI continues to focus on the goals of our mission to provide a diverse community of bench to bedside research scientists the fundamental biological datasets needed to better understand the human condition from the many ways that mice model normal and pathologic human biology. Looking forward, we anticipate progress on several fronts. Our genome feature catalog is upgraded regularly, and we expect to continue expanding representation of predicted and verified regulatory features as they are characterized. Improvements to the mouse Y chromosome assembly and its annotations will fill in a number of Chr Y features missing from the catalog and will allow us to extend our representation of PARX/PARY partners. We will also add new relationships between feature types to capture experimentally verified cases of regulatory region features acting to influence the expression of mouse genes. We will continue expanding our index of mouse strain variants by loading SNP data from the Wellcome Sanger Mouse Genomes Project (MGP) (Keane *et al.* 2011), and will follow that by loading the comparatively expansive MGP indel set. We are also preparing for the integration of genome-scale mouse structural variants, now that long-read assemblies of mouse strain genomes are starting to emerge and for the first time are providing genome-wide resolution of structural variants (Ferraj *et al.* 2023). We are currently upgrading MGI infrastructure to better manage user interface performance with such large datasets.

This infrastructure boost will also enable us to load additional bulk-RNA-Seq data, to expand GXD to include scRNA-Seq data, and to further augment expression search capabilities. Current GXD differential and expression profile searches will be enhanced by including RNA-Seq data. RNA-Seq data will vastly improve the reliability of “not expressed in” searches, as every gene is assayed in each sample.

To facilitate visualization of variation between genomes, upgrades to the MGV are planned. The MGV will be updated to obtain paralog information directly from the Alliance (in addition to ortholog information), replacing the MGV's current method of inferring paralogs between genomes from shared orthology (Richardson *et al.* 2022). Other changes planned for the MGV are to support, (1) comparisons of the detailed structure of individual genes across different genomes, (2) uploading private annotated genomes, and (3) the integration of other data types, such as different classes of variants. To complement MGI variant resources, we are exploring options for leveraging the wealth of mouse genome variant data represented in GenomeMUSter, a variant data service that employs phylogeny-informed imputation to fill in missing SNP genotypes across 657 mouse strains (Ball *et al.* 2024). To further assist the display and usability of the massive volume of interstrain variation, we are also looking into how MGI can support efforts to develop a reference mouse pangenome, as has recently been drafted for human (Eisenstein 2023; Liao *et al.* 2023).

Working with our collaborators at the IMPC and the Jackson Laboratory, we will continue to maintain a comprehensive catalog of mouse mutant alleles and will regularly update phenotype information as it becomes available from IMPC-affiliated phenotyping centers. In addition, we will update mutation descriptions for endonuclease-mediated (CRISPR-Cas9) alleles to include the precise (BLAT-aligned) genome coordinate breakpoints of engineered deletions. We continue to standardize mouse mutation annotations by transforming text-based descriptions of literature-curated variants into sequence variant nomenclature based on Human Genome Variation Society (HGVS) notation. We will also proceed to synergize phenotype concepts and terms between mouse and human to enhance cross-species phenotype searching in the HMDC.

As a founding member of the Alliance, we share the vision that aggregation and congruence of knowledge between model organisms leads to a resource that can exceed the sum of its parts. We continue efforts to harmonize components of MGI infrastructure with Alliance Central infrastructure, as components and workflows conform, with the aim of centralizing these infrastructure components at the Alliance. As the premier knowledgebase of genetic and genomic science of the laboratory mouse, MGI remains a critical source of interconnected mouse and human information for the research interests of experimental and computational scientists who study the mouse as an important part of the collective pursuit of improved human health.

## Methods

### Automating literature acquisition and triage

Transitioning our literature acquisition and triage processes to automation started with full text extraction from PDFs, populating citation metadata from PubMed, and implementing keyword searches to serve our secondary review screening process, which was still a manual process at the time. We then began automated reference acquisition with full text search and download of PDFs for a few journals from PubMed Central and refined our text extraction step to split and store article text in useful subsections for curation, including separating the reference section from other text to improve the article-relevant keyword searching used for secondary review. Recent improvements have fully automated the MGI relevance decision by incorporating a supervised machine learning step (that classifies papers as “keep” or “discard”) on full extracted text (minus references), and by implementing improved curation-group-specific secondary screening to automatically direct classifier predicted MGI-relevant (keep) publications to appropriate curation groups, based on the types of data presented in the papers.

Most recently, we added Science Direct to our automated PDF downloads and now access articles from 153 journals (94 from PubMed Central and 59 from Science Direct). Most journals in our core journal set are now accessible this way, but there remain a few journals that currently do not provide open access downloads, and we still monitor those journals manually. Automating the MGI relevance step allows us to use a broad full text search for PDFs (containing the word “mice”), which makes our downloads likely to include all potentially relevant articles. Our automated literature acquisition pipeline brings in more total papers per year now, due to automated reference retrieval and less stringent criteria for reference entry. The MGI relevance classifier also records significantly more MGI-relevant papers (keeps) per year, compared to when MGI relevance evaluation was manual, and criteria for reference entry was more stringent. Consequently, MGI curators can devote more time to reference indexing and full reference data entry.

### Data discrepancy and retraction handling

MGI reports all conflicting results from literature or loaded resources without prejudice, and allows users to assess these results. However, in some cases of obvious error, we do contact authors or data providers for clarification or correction, and state if any changed data are from an author correction. Most data from withdrawn or fully retracted papers are removed from the system unless an author validates the information.

### Quick search update

Technical improvements to the Quick Search include performance gains by converting the search engine to Apache Solr and simplifying the indexes. Better result ranking was accomplished for each of the five tabs by incorporating tab-specific data type weights and match quality priority boosts.

### MGV update

Technical improvements to the MGV include incorporating the standard indexing methods tabix and faidx to access GFF and FASTA data at runtime, which are more performant and robust than the custom file formats used by the original version. The data preparation system has been streamlined, extending and customizing the build is easier now, and configuration is now based on YAML, which is much more human-friendly than the previous JSON configs. Lastly, the Vue.js JavaScript framework that the Viewer is implemented with has been upgraded to the latest version (Vue 3).

### MGI feature types

MGI uses a two-tiered annotation system to represent genome feature types (Table 2). All genome features have a general primary feature type, and some primary feature types have more descriptive subcategories. Users can view the full list of feature types and search by feature type hierarchically from the MGI Genes and Markers Query Form (https://www.informatics.jax.org/marker) (not shown). On gene detail pages, the primary feature type is displayed in the page title, while the feature type subcategory (if present) is shown as the Feature Type in the Summary section (see Fig. 3). If the primary feature type has no subcategories, then the primary type is displayed as feature type (as for QTL, for example). In assigning feature types, we follow the Sequence Ontology (SO) as much as possible. A complete list of SO terms used in MGI, and their definitions, is available (https://www.informatics.jax.org/userhelp/GENE_feature_types_help.shtml).

### Relationships between genome features

#### Transcription Start Sites

Mouse Transcription Start Site (TSS) features in MGI are part of a one-time load from the FANTOM Consortium (Functional ANnoTation Of the Mammalian genome) as part of the FANTOM5 project (Fantom Consortium *et al.* 2014). FANTOM5 TSS elements are clusters of CAGE peaks (Cap Analysis of Gene Expression), which potentially overlap functional promoters within the 399 mouse samples assayed in the study. We represent FANTOM5 TSS elements as distinct genome features with sequentially numbered nomenclature that follows structure: symbol = Tssr#, name = transcription start site region #. Each TSS feature has genome coordinates updated to Build 39 using NCBI's Remap tool. The average size of TSS features (CAGE clusters) is 21 bp. Without experimental verification that transcripts for a specific gene initiate within TSS features, we do not establish equivalence between these elements and MGI genes. However, it is useful to know that a given gene has TSS elements nearby, thus we establish relationships between TSS features and genes using a rule-based association process that considers proximity of TSS features to the 5′ ends of genes. If a TSS feature is within 2 kb upstream (or within coordinates) of one or more MGI genes, we establish a TSS relationship to the gene with the closest 5′-end to the TSS midpoint. We associate TSS features with at most one MGI gene (many have no gene association) and allow MGI genes to be associated with any number of TSS features, since genes can have multiple promoters.

The number of Transcription Start Site (TSS) features associated with a gene is shown in the lower right corner of gene detail page Summary section (Fig. 3). Clicking the TSS count opens a popup table containing details about each associated TSS feature, including symbol, coordinates, and the distance each TSS is located from the associated gene (upstream or downstream) (not shown). Links are provided in the TSS popup table to detail pages for the associated TSS genome features and to a view of the associated gene in genome context from the JBrowse genome browser. The *Pten* gene, for example, has 18 associated TSS features (Fig. 3) that span a wide range of coordinates within the gene (not shown). The “Transcription Start Site” subsection on gene detail pages is reciprocal between genes and the corresponding TSS features. On TSS detail pages, this subsection displays “Transcription Start Site for” and lists the associated gene with a reciprocal link to the gene's detail page (not shown). Also displayed is the distance between the TSS feature and the 5'-end of the associated gene (upstream or downstream).

#### Candidate for QTL

If a gene is a reported candidate for one or more QTL, information about the QTL is provided in a new “Candidate for QTL” category of the gene detail Summary section, using a similar display model as for TSS features (Fig. 3). The number of QTL for which the gene is a candidate is shown, and clicking on the count opens a popup table with more details about the QTL, including links to QTL detail pages and to supporting references. The *Pten* gene is a candidate for the *Thcir3* QTL (not shown). On detail pages of corresponding QTL (also not shown), the same page area displays a reciprocal subsection: “Candidate Genes”, where the number of candidate genes reported for the QTL is indicated, and links to a Candidate Genes popup table with additional information and links for the candidate genes.

#### Interacting QTL

If a QTL has reported genetic interactions with other QTL, information about the QTL interactions is provided on QTL detail pages in a new “QTL Interactions” subsection, which appears in the same page area as the “Candidate for QTL” subsection on gene detail pages (described above) (Candidate for QTL not shown). The “QTL Interactions” subsection lists the number of interacting QTL and has a link to a popup table providing additional information about that QTL's interactions, including interaction types, links to interacting QTL details and to supporting references.

### Genomic and genetic map locations

For features with genome coordinates, the reference genome coordinates are derived either from the feature's representative genomic sequence (see *Sequences & Gene Models* section) or are provided by other sources (see *Assigning reference genome coordinates to genome features* section). Genetic map information in MGI is derived from literature-directed MGI curator updates of the discontinued mouse Chromosome Committee Reports. Mouse genetic map data are published infrequently now, in this age of whole-genome science.

### Sequences & Gene Models

MGI loads information for over 13 million mouse sequences from major sequence providers (NCBI, Ensembl and UniProt), and connects sequences to genes and genome features through a series of quality-controlled association loads and through curation. Novel mouse sequence accession IDs and source information are loaded weekly from GenBank, RefSeq and UniProt. Mouse gene model information is updated from Ensembl and NCBI regularly, as new annotation builds become available. MGI is currently using NCBI gene model annotation build NCBI_RS_2023_04, and Ensembl build 111.

### Representative sequence selection

Choosing the representative genome sequence is central to the process, as the representative transcript and protein sequences depend on a gene's representative genomic sequence. Gene model sequences from Ensembl, NCBI, and VISTA annotation loads are prioritized for representative genomic sequence selection. In MGI, gene model sequences represent the genomic region defined by the start/end coordinates from a provider, including the sequence regions defined by providers of regulatory region features. For MGI genes and pseudogenes with coordinates, intron/exon boundary information can be found in the mgi_gff3 file (https://www.informatics.jax.org/downloads/mgigff3/MGI.gff3.gz).

#### Representative genomic sequence

For protein-coding and noncoding RNA genes and pseudogenes, we select either the Ensembl or NCBI gene model as the representative genomic sequence. If a feature has gene models from both providers, then we select the shortest of the two, to avoid cases where one provider has included an extended read-through transcript in their gene model. If there are no associated gene models, we select the longest associated GenBank genomic sequence. For regulatory region features, we select the Ensembl, NCBI or VISTA gene model as representative genomic sequence. For enhancers that have both NCBI and VISTA gene models, the NCBI model is selected as representative.

#### Representative transcript and protein sequences

The representative transcript and protein sequences for a genome feature are selected algorithmically, based on the feature's representative genomic sequence. If the representative genomic is from Ensembl, then we choose the longest Ensembl protein and corresponding transcript as representatives. If the representative genomic is not from Ensembl, then we select the longest transcript from the provider of the genomic gene model as the representative transcript and, if coding, we select as representative protein the longest associated protein from a provider hierarchy. If the representative genomic is not a gene model from an annotation provider, then both representative transcript and protein (if coding) sequences are selected from provider hierarchies [Transcript hierarchy: (longest of NM RefSeq > NR RefSeq > GenBank non-EST RNA > XM RefSeq > XR RefSeq > GenBank EST RNA); Protein hierarchy: (longest of SWISS-PROT > RefSeq NP > TrEMBL > RefSeq XP)].

### Assigning reference genome coordinates to genome features

If a genome feature is associated with one or more gene model sequences, then one of these gene models will be selected as the feature's representative genomic sequence following the rules described above. In these cases, the genome coordinates of the representative genomic sequence serve as the coordinates of the associated genome feature. For genome features that have no associated gene models but have associated GenBank sequences (genomic and/or transcript), MGI uses a BLAT alignment pipeline to select coordinates for the feature. The BLAT pipeline selects all high-quality sequences associated with participating features and automates BLAT alignments against the current reference mouse genome assembly using MGI's dedicated BLAT server. Genome coordinates are assigned to the feature from the longest high-quality alignment. All genome feature coordinates derived from the MGI BLAT pipeline are reviewed and approved by MGI curators, and the coordinate provider listed for these coordinates is MGI. For most feature types in MGI, genome coordinates are derived either from gene model sequences or from sequences used in BLAT alignments. For some feature types (TSS features and CpG Islands), genome coordinates were part of the original genome feature loads and have been updated to GRCm39 using NCBI's remap tool. The coordinate provider in these cases is also listed as MGI.

### Build 39 update

Fully transitioning from Build 38 (GRCm38) to Build 39 (GRCm39) coordinates for the reference mouse genome assembly was an orchestrated, sequential, multistep process. MGI obtains genome coordinates for genes, pseudogenes and other genome features of biological relevance from a variety of sources. For genes and pseudogenes, we obtained Build 39 coordinates from updated NCBI and Ensembl gene models or by BLAT-aligning literature-curated GenBank sequences to the Build 39 assembly. Then, seeded with MGI gene-to-sequence ID associations from our Build38 mgi.gff3 file (with updated Build 39 coordinates for those sequence IDs), we ran our gene unification process, which computes genome coordinate overlaps and generates distinct gene-to-sequence ID unions for each gene and pseudogene (Zhu *et al.* 2015). This process was simplified because gene-to-sequence ID associations change very little between mouse annotation builds (from NCBI and Ensembl) resulting in relatively few cases of novel or obsolete genes (which were curated separately and then incorporated into the next round of gene unification). For other genome features with Build 38 coordinates (transcription start sites, CpG Islands, transgene insertions, QTL, STS markers, etc.), we used NCBI's Remap resource to convert Build 38 coordinates to Build 39. MGI also stores genome coordinates for gene trap alleles and allele variants, and genome coordinates appear in several note fields, all of which were updated to Build 39 coordinates using NCBI's remap resource.

### Pseudoautosomal region representation

MGI's representation of PARX/PARY features as separate genes raised the question of which annotations (phenotype, expression, GO, etc.) associated with either the PARX or PARY gene should be shared with the partner gene. We have chosen to share only NCBI Gene IDs and UniProt IDs between PARX/PARY partners initially. As the PAR region in mouse becomes better characterized, we will have a better sense of the incidence of heteroallelism in the PAR, which could influence the extent to which we share annotations between PARX/PARY partners. The provisional report of a more complete C57BL/6J PAR assembly (Kasahara *et al.* 2022) describes copy-number polymorphism between PARX and PARY of a segmental duplication containing two truncated PAR genes that starts in the Y-unique region near the PAR boundary and extends into the PAR for ∼85 kb. Such genetic variation between PARX and PARY regions could give rise to PARX- and PARY-specific phenotypes and/or expression patterns. As mentioned, MGI's representation of PARX/PARY partners is well suited to accommodate such potential heterogeneity.

### Regulatory feature equivalence

We do not attempt to determine equivalence between Ensembl and VISTA or NCBI RefSeqFEs regulatory features by genome coordinate overlap (as we do for Ensembl and NCBI gene features; Zhu *et al.* 2015) due to differences in regulatory feature prediction strategies. A number of NCBI enhancer features (∼600) cross-reference VISTA enhancers and have identical coordinates, thus we considered these equivalent genome features, and associated corresponding VISTA and NCBI identifiers and gene models with them. Links are provided from the Location & Maps section of regulatory feature detail pages to corresponding regions in the JBrowse genome browser for genome context, and separate JBrowse tracks for the ENSEMBL Regulatory Build and VISTA Enhancers can be toggled on to view other regulatory features in that genomic region.

### SNP-to-genome feature associations

SNPs located within the coordinates of genome features were annotated as “within coordinates of” the associated features. Associations to genome features were extended to include SNPs within 10 kb of the associated feature (upstream or downstream). These SNPs were annotated as “within distance of” the associated features, with the bp distance and direction from the feature stored in the database and displayed in the user interface. SNPs can have associations with any number of genome features but can have only a single association with a given genome feature (“within distance of” or “within distance of”).

#### Incomplete SNP function classifications

The SNPs associated with *Tyr* in Fig. 7f list “within coordinates of” as their function classification (Category). This annotation is incomplete. We know, for example, that rs31191169 represents a missense variant in the *Tyr* gene. After converting SNP coordinates to Build 39, our SNP annotations were limited to within coordinates of genes and to the distance (in bp) upstream or downstream of genes (10,000 bp max), since we no longer have corresponding updated functional classification annotations from dbSNP. We are reconfiguring our SNP load to process SNPs from other sources, such as the Wellcome Sanger MGP (Keane *et al.* 2011) and the European Variation Archive (Cezard *et al.* 2022), and will update SNP function classifications in conjunction with our updated SNP load. The MGP SNP set alone will increase the total number of SNPs in MGI by over 4-fold and the number of strain alleles by about 20-fold.

### Community data submissions

MGI accepts user data submissions for gene or marker, allele, strain, phenotype, expression and recombinase activity (https://www.informatics.jax.org/submit.shtml). Different submission forms for each data type are available. Requested new gene, allele/mutation or strain symbols may be reserved in advance of publication, with authors receiving an MGI ID for the new symbols. Reserved nomenclature and other data are kept private until publication unless the user requests that the information be made public at the time of submission or thereafter.

## Supplementary Material

iyae031_Supplementary_Data

## Data Availability

MGI data are freely available through a variety of outlets. The primary means of access is our public web interface at https://www.informatics.jax.org, shown in many examples throughout this paper. Additional mechanisms include downloadable public data reports (https://www.informatics.jax.org/downloads/reports/index.html); MouseMine, featuring a robust web services API (https://www.mousemine.org); and online SQL accounts that provide programmatic access to a read-only copy of the database. See the MGI developer's tools page for details on how to access these services (https://www.informatics.jax.org/software.shtml). MGI data are updated weekly. The date of the latest refresh is found at the bottom of every page. MGI software updates occur several times per year. The software release number is also found at the bottom of every page. Supplemental material is available at GENETICS online.

## Mouse Genome Informatics Group

Anna Anagnostopoulos, Dale A. Begley, Susan M. Bello, Karen Christie, Jacqueline H. Finger, Paul Hale, Terry F. Hayamizu, David P. Hill, Michelle N. Knowlton, Debra M. Krupke, Monica McAndrews, Meiyee Law, Ingeborg J. McCright, Li Ni, Hiroaki Onda, Dmitry Sitnikov, Constance M. Smith, Monika Tomczuk, Laurens Wilming, Jingxia Xu, Yunxia Zhu, Olin Blodgett, Jeffrey W. Campbell, Lori E. Corbani, Peter Frost, Sharon C. Giannatto, David B. Miers, Howie Motenko, Steven B. Neuhauser, David R. Shaw, Nancy E. Butler, and Janice E. Ormsby.
