# Supplementary material for: Mouse Genome Informatics: an integrated knowledgebase system for the laboratory mouse
Source: Genetics. 2024 Mar 26;227(1):iyae031. doi: 10.1093/genetics/iyae031 (PMC11075557; doi:10.1093/genetics/iyae031)
Supplement: iyae031_Supplementary_Data [file iyae031_supplementary_data.zip › Figure_S1_GENETICS-2023-306303.pdf]

Mouse Genome Informatics (MGI): An integrated knowledgebase system for the laboratory mouse

Richard M. Baldarelli, Cynthia L. Smith, Martin Ringwald, Joel E. Richardson, Carol J. Bult, Mouse Genome Informatics Group

The Jackson Laboratory, Bar Harbor, ME 04609, USA

Figure S1

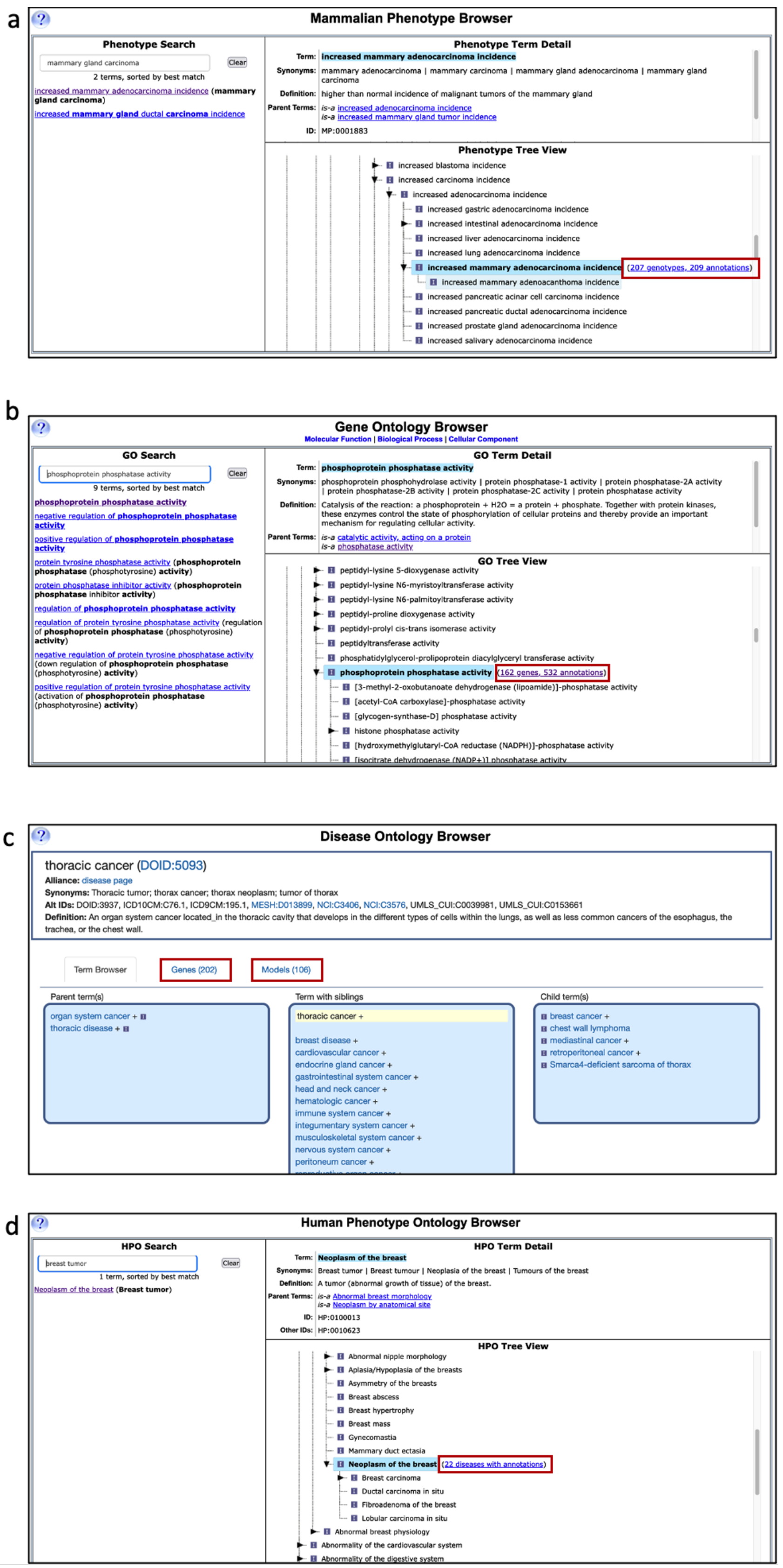

Figure S1. Examples of Ontology Browsers in MGI. MGI maintains ontology browsers for anatomy, phenotype and disease, and gene function. Shown are four examples of the browsers in MGI. (a) The Mammalian Phenotype Ontology Browser, (b) The Gene Ontology Browser, (c) The Disease Ontology Browser, and (d) The Human Phenotype Ontology Browser. Each browser shows the ontology terms and the relationship between the terms. Each term is linked to data within MGI for easy access to data of interest.
