# Supplementary material for: Mouse Genome Informatics: an integrated knowledgebase system for the laboratory mouse
Source: Genetics. 2024 Mar 26;227(1):iyae031. doi: 10.1093/genetics/iyae031 (PMC11075557; doi:10.1093/genetics/iyae031)
Supplement: iyae031_Supplementary_Data [file iyae031_supplementary_data.zip › Figure_S2_GENETICS-2023-306303.pdf]

Mouse Genome Informatics (MGI): An integrated knowledgebase system for the laboratory mouse

Richard M. Baldarelli, Cynthia L. Smith, Martin Ringwald, Joel E. Richardson, Carol J. Bult, Mouse Genome Informatics Group

The Jackson Laboratory, Bar Harbor, ME 04609, USA

Figure S2

Vertebrate Homology

Source	Alliance of Genome Resources

Comparative GO Graph (mouse, human, rat, zebrafish) Multiple Genome Viewer Alliance Homology Information

Species	Symbol	Gene Links	Genetic Location	Genome Coordinates (mouse and human only)	Associated Human Diseases	Sequences select all deselect all get FASTA Go
human	PTEN	HGNC:9588 (HGNC)<br>5728 (Entrez Gene)<br>601728 (OMIM)<br>PTEN (Alliance of Genome Resources)	Chr10 q23.31	Chr10:87862563-87971930 (+)<br>GRCh38	Bannayan-Riley-Ruvalcaba syndrome<br>breast cancer<br>esophageal cancer<br>familial meningioma<br>high grade glioma<br>lung non-small cell carcinoma<br>macrocephaly-autism syndrome<br>ovarian cancer<br>peripheral nervous system neoplasm<br>prostate cancer<br>PTEN hamartoma tumor syndrome<br>sporadic breast cancer<br>urinary bladder cancer	☐ P60484 (UniProt | EBI)<br>☐ NM_000314 (RefSeq)
mouse	Pten	MGI:109583 (MGI)<br>19211 (Entrez Gene)<br>Gene Tree<br>Pten (Alliance of Genome Resources)	Chr19 28.14 cM	Chr19:32734977-32803560 (+)<br>GRCm39	acute lymphoblastic leukemia<br>autism spectrum disorder<br>Bannayan-Riley-Ruvalcaba syndrome<br>brain disease<br>Cowden syndrome<br>endometrial cancer<br>fatty liver disease<br>hepatocellular carcinoma<br>intestinal pseudo-obstruction<br>macrocephaly-autism syndrome<br>persistent fetal circulation syndrome<br>prostate cancer<br>thyroid gland follicular carcinoma<br>urinary bladder cancer	☐ 19211 (NCBI Gene Model)<br>☐ O08586 (UniProt | EBI)<br>☐ NM_008960 (RefSeq)
rat	Pten	RGD:61995 (Rat Genome Database)<br>50557 (Entrez Gene)<br>Pten (Alliance of Genome Resources)	Chr1 q52			☐ O54857 (UniProt | EBI)<br>☐ NM_031606 (RefSeq)
zebrafish	ptena	ZDB-GENE-030131-3776 (Zebrafish Model Organism Database)<br>794088 (Entrez Gene)<br>ptena (Alliance of Genome Resources)	Chr17			☐ NP_957002 (RefSeq)<br>☐ NM_200708 (RefSeq)
	ptenb	ZDB-GENE-030616-47 (Zebrafish Model Organism Database)<br>368415 (Entrez Gene)<br>ptenb (Alliance of Genome Resources)	Chr12			☐ NP_001410617 (RefSeq)<br>☐ NM_001001822 (RefSeq)

Figure S2. The Vertebrate Homology Page for *Pten*. MGI obtains stringent ortholog assertions from the Alliance of Genome Resources. The MGI Vertebrate Homology table for mouse *Pten* lists the mouse gene with orthologs from human, rat and zebrafish (zebrafish has two *Pten* orthologs, *ptena* and *ptenb)*. Links are provided to corresponding records at Entrez Gene (NCBI Gene), the Alliance, and at the relevant organism databases for all genes in the table. A Gene Tree link is also provided for the mouse gene. Chromosome and map location (if available) are provided for all orthologs in the table, while genome coordinates are provided for the mouse and human orthologs. Also shown for mouse and human orthologs are associated human diseases, with links to the MGI Disease Ontology Browser. The last column shows selective transcript and protein sequence IDs for each ortholog (accession ID-to-gene associations obtained from UniProt and NCBI), and the representative genomic sequence ID for the mouse gene. FASTA sequences are available selected sequences, or the selected sequences can be forwarded to NCBI BLAST (not shown). Above the table, links are provided to Comparative Gene Ontology Graphs between orthologs in the table, and to the Multiple Genome Viewer aligned on *Pten*, with the genomes for mouse (C57BL/6J), human, and rat rendered. The URL for this page is: https://www.informatics.jax.org/homology/cluster/key/45855675 and is accessible from the Pten gene detail page in the Homology section, under MGI Vertebrate Homology: Pten stringent orthology.
