# Supplementary material for: Mouse Genome Informatics: an integrated knowledgebase system for the laboratory mouse
Source: Genetics. 2024 Mar 26;227(1):iyae031. doi: 10.1093/genetics/iyae031 (PMC11075557; doi:10.1093/genetics/iyae031)
Supplement: iyae031_Supplementary_Data [file iyae031_supplementary_data.zip › Figure_S3_GENETICS-2023-306303.pdf]

Mouse Genome Informatics (MGI): An integrated knowledgebase system for the laboratory mouse

Richard M. Baldarelli, Cynthia L. Smith, Martin Ringwald, Joel E. Richardson, Carol J. Bult, Mouse Genome Informatics Group

The Jackson Laboratory, Bar Harbor, ME 04609, USA

Figure S3

$Rr20^{tm1.1Vlcg}$

Targeted Allele Detail

Your Input Welcome

Summary | Mutation origin | Mutation description | Expression | Phenotypes | Disease models | Find Mice (IMSR) | References

Summary

Symbol: $Rr20^{tm1.1Vlcg}$
Name: regulatory region 20; targeted mutation 1.1, Velocigene
MGI ID: MGI:5441845
Synonyms: ECR5⁻
Gene: Rr20 *Location:* unknown *Genetic Position:* Chr11, Syntenic
Alliance: $Rr20^{tm1.1Vlcg}$ page

Higher bone mass in $Rr20^{tm1.1Vlcg}/Rr20^{tm1.1Vlcg}$ and $Sost^{tm1(KOMP)Vlcg}/Sost^{tm1(KOMP)Vlcg}$ mice

Show the 1 phenotype image(s) involving this allele.

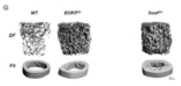

Mutation origin

Germline Transmission: Earliest citation of germline transmission: J:188586
Parent Cell Line: F1H4 (ES Cell)
Strain of Origin: (129S6/SvEvTac x C57BL/6NTac)F1

Mutation description

Allele Type: Targeted (Modified regulatory region, Null/knockout)
Mutations: Insertion, Intergenic deletion

Mutation details: The evolutionarily conserved candidate enhancer element (ECR5) that acts as a distal enhancer to Sost was replaced with a floxed neo cassette. Cre-mediated recombination removed the neo cassette. Immunohistochemistry confirmed highly variable expression of Sost. (*J:188586*)

$Rr20^{tm1.1Vlcg}/Rr20^{tm1.1Vlcg}$ mice have HBM due to reduced number of Sost-expressing osteocytes

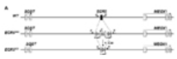

Phenotypes

Key:

hm	homozygous	ht	heterozygous	tg	involves transgenes	√	phenotype observed
cn	conditional genotype	cx	complex: > 1 genome feature	ot	other: hemizygous, indeterminate,…	N	normal phenotype

Genotype/ Background:

		Allelic Composition	Genetic Background	Cell Line(s)
hm1	*Disease Model*	$Rr20^{tm1.1Vlcg}/Rr20^{tm1.1Vlcg}$	involves: 129S6/SvEvTac * C57BL/6NTac	

Phenotypes:

**Affected Systems** (show or hide all annotated terms)	hm1
**skeleton**	√
skeleton phenotype	N
abnormal osteoblast morphology	√
abnormal trabecular bone morphology	√
increased trabecular bone volume	√
increased bone trabecula number	√
increased trabecular bone connectivity density	√
increased bone mass	√
abnormal bone ossification	√
abnormal bone mineralization	√

View phenotypes and curated references for all genotypes (concatenated display).

Disease models

Key: √ disease model | ⊘ expected model not found

Models:

**Human Diseases**		hm1
SOST-related sclerosing bone dysplasia	IDs	√

Expression

In Structures Affected by this Mutation: 2 anatomical structures

Find Mice (IMSR)

Mouse strains and cell lines available from the International Mouse Strain Resource (IMSR)

Carrying this Mutation: Mouse Strains: 0 strains available Cell Lines: 0 lines available
Carrying any Rr20 Mutation: 0 strains or lines available

References

Original: J:188586 Collette NM, et al., Targeted deletion of Sost distal enhancer increases bone formation and bone mass. Proc Natl Acad Sci U S A. 2012 Aug 28;109(35):14092-7
All: 2 reference(s)

Figure S3. Regulatory Region Allele detail page for $Rr20^{tm1.1Vlcg}$. Alleles for regulatory region markers are shown in MGI and associated with the regulatory region marker in MGI. Summary description and associated information are like that on other allele detail pages, including molecular details, summary phenotype and disease model information and references.
