# Supplementary material for: Mouse Genome Informatics: an integrated knowledgebase system for the laboratory mouse
Source: Genetics. 2024 Mar 26;227(1):iyae031. doi: 10.1093/genetics/iyae031 (PMC11075557; doi:10.1093/genetics/iyae031)
Supplement: iyae031_Supplementary_Data [file iyae031_supplementary_data.zip › Figure_S4_GENETICS-2023-306303.pdf]

Mouse Genome Informatics (MGI): An integrated knowledgebase system for the laboratory mouse

Richard M. Baldarelli, Cynthia L. Smith, Martin Ringwald, Joel E. Richardson, Carol J. Bult, Mouse Genome Informatics Group

The Jackson Laboratory, Bar Harbor, ME 04609, USA

Figure S4

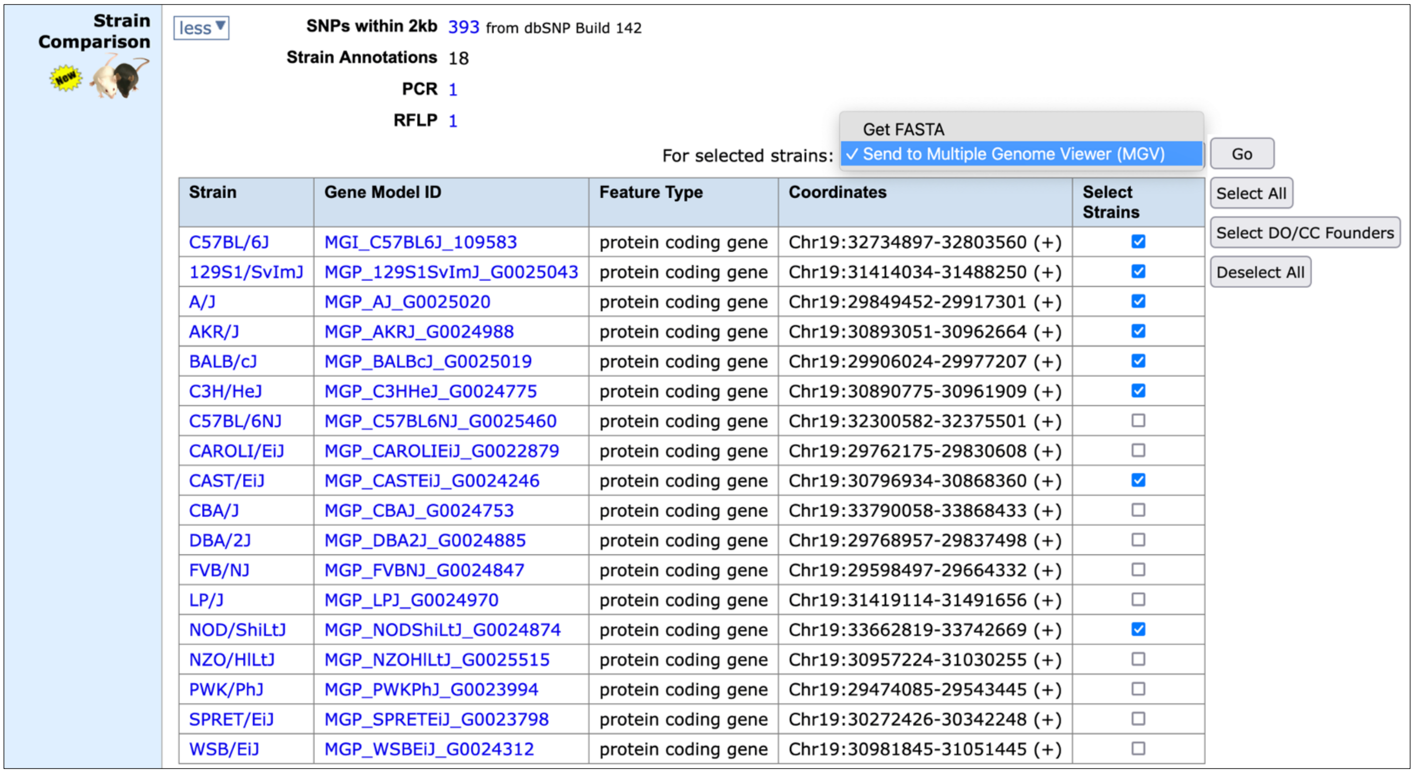

Strain	Gene Model ID	Feature Type	Coordinates	Select Strains
C57BL/6J	MGI_C57BL6J_109583	protein coding gene	Chr19:32734897-32803560 (+)	☑
129S1/SvImJ	MGP_129S1SvImJ_G0025043	protein coding gene	Chr19:31414034-31488250 (+)	☑
A/J	MGP_AJ_G0025020	protein coding gene	Chr19:29849452-29917301 (+)	☑
AKR/J	MGP_AKRJ_G0024988	protein coding gene	Chr19:30893051-30962664 (+)	☑
BALB/cJ	MGP_BALBcJ_G0025019	protein coding gene	Chr19:29906024-29977207 (+)	☑
C3H/HeJ	MGP_C3HHeJ_G0024775	protein coding gene	Chr19:30890775-30961909 (+)	☑
C57BL/6NJ	MGP_C57BL6NJ_G0025460	protein coding gene	Chr19:32300582-32375501 (+)	☐
CAROLI/EiJ	MGP_CAROLIEiJ_G0022879	protein coding gene	Chr19:29762175-29830608 (+)	☐
CAST/EiJ	MGP_CASTEiJ_G0024246	protein coding gene	Chr19:30796934-30868360 (+)	☑
CBA/J	MGP_CBAJ_G0024753	protein coding gene	Chr19:33790058-33868433 (+)	☐
DBA/2J	MGP_DBA2J_G0024885	protein coding gene	Chr19:29768957-29837498 (+)	☐
FVB/NJ	MGP_FVBNJ_G0024847	protein coding gene	Chr19:29598497-29664332 (+)	☐
LP/J	MGP_LPJ_G0024970	protein coding gene	Chr19:31419114-31491656 (+)	☐
NOD/ShiLtJ	MGP_NODShiLtJ_G0024874	protein coding gene	Chr19:33662819-33742669 (+)	☑
NZO/HlLtJ	MGP_NZOHlLtJ_G0025515	protein coding gene	Chr19:30957224-31030255 (+)	☐
PWK/PhJ	MGP_PWKPhJ_G0023994	protein coding gene	Chr19:29474085-29543445 (+)	☐
SPRET/EiJ	MGP_SPRETEiJ_G0023798	protein coding gene	Chr19:30272426-30342248 (+)	☐
WSB/EiJ	MGP_WSBEiJ_G0024312	protein coding gene	Chr19:30981845-31051445 (+)	☐

Figure S4. Strain Comparison Section of the *Pten* Gene Detail Page. The toggled-open state of the Strain Comparison section shows counts of SNPs and Strain Annotations (retained from the closed state) along with counts of PCR and RFLP variants, all of which link to more details. The strain comparison table (only viewed in the toggled-open state) lists 18 fully sequenced and annotated mouse strains, including the reference C57BL/6J strain, 16 additional mouse inbred strains (KEANE *et al.* 2011; LILUE *et al.* 2018) and the wild-derived *Mus caroli* strain CAROLI/EiJ (THYBERT *et al.* 2018). Included in the table are links to MGI strain detail pages for corresponding strains, links to MGI sequence detail pages for corresponding strain-specific *Pten* gene models, and corresponding feature types and strain-specific genome coordinates for each gene model. The strain-specific gene model for C57BL/6J is derived from the outermost boundary coordinates of a union of the gene model annotations from Ensembl and NCBI for *Pten*. The Select Strains column allows selection of one or more strains for forwarding (Go button) to the Multiple Genome Viewer (aligned on *Pten*, with the selected strain genomes rendered) or to obtain corresponding gene model FASTA sequences. Other strain selection options are provided to select or deselect all strains, or to select the 8 founder strains of the Diversity Outbred (DO) and Collaborative Cross (CC) initiatives (BOGUE *et al.* 2015).
