# Supplementary material for: Mouse Genome Informatics: an integrated knowledgebase system for the laboratory mouse
Source: Genetics. 2024 Mar 26;227(1):iyae031. doi: 10.1093/genetics/iyae031 (PMC11075557; doi:10.1093/genetics/iyae031)
Supplement: iyae031_Supplementary_Data [file iyae031_supplementary_data.zip › Figure_S5_GENETICS-2023-306303.pdf]

Mouse Genome Informatics (MGI): An integrated knowledgebase system for the laboratory mouse

Richard M. Baldarelli, Cynthia L. Smith, Martin Ringwald, Joel E. Richardson, Carol J. Bult, Mouse Genome Informatics Group

The Jackson Laboratory, Bar Harbor, ME 04609, USA

Figure S5

Human Disease		Mouse Models
Bannayan-Riley-Ruvalcaba syndrome	IDs	View 3 models
macrocephaly-autism syndrome	IDs	View 2 models
prostate cancer	IDs	View 20 models
urinary bladder cancer	IDs	View 3 models
acute lymphoblastic leukemia	IDs	View 1 model
autism spectrum disorder	IDs	View 3 models
brain disease	IDs	View 1 model
Cowden syndrome	IDs	View 12 models
endometrial cancer	IDs	View 3 models
fatty liver disease	IDs	View 2 models
hepatocellular carcinoma	IDs	View 3 models
intestinal pseudo-obstruction	IDs	View 1 model
persistent fetal circulation syndrome	IDs	View 1 model
thyroid gland follicular carcinoma	IDs	View 3 models
breast cancer	IDs	View 5 models
esophageal cancer	IDs	
familial meningioma	IDs	
high grade glioma	IDs	View 5 models
lung non-small cell carcinoma	IDs	
ovarian cancer	IDs	View 8 models
peripheral nervous system neoplasm	IDs	
PTEN hamartoma tumor syndrome	IDs	
sporadic breast cancer	IDs	

Click on a disease name to see all genes associated with that disease.

Figure S5. Summary Human Disease and Mouse Models Table. Shown is the open human disease summary table for known human diseases associated with the human PTEN gene and mouse models annotated to these diseases.
